# From single-pathway cascade to network pathophysiology: how genetically engineered mouse models reshaped our understanding of pancreatitis

**DOI:** 10.3389/fphys.2026.1924373

**Published:** 2026-08-19

**Authors:** Ting Yan, Qinghua Lin, Shaoqun Huang, Lingxiang Wang

**Affiliations:** 1 Department of General Surgery, Second Affiliated People’s Hospital, Fujian University of Traditional Chinese Medicine, Fuzhou, China; 2 Fujian Provincial Governmental Hospital, Fuzhou, China; 3 Department of General Surgery, Hunan Cancer Hospital/The Affiliated Cancer Hospital of Xiangya School of Medicine, Central South University, Changsha, China

**Keywords:** genetically engineered mouse models, network pathophysiology, pancreatitis, regulated cell death, trypsinogen activation

## Abstract

Pancreatitis remains a common gastrointestinal disease with no mechanism-specific therapy. Its pathogenesis was long explained by the autodigestion hypothesis: a single linear cascade initiated by premature intra-acinar trypsinogen activation. This trypsin-centric view cannot account for the amplification of inflammation, the switching of cell-death modality, the acute-to-chronic transition, organ failure, or the chronic pancreatitis caused by protein-misfolding genes that act outside the protease system. Here we synthesize evidence from genetically engineered mouse models (GEMMs); their loss- and gain-of-function designs, spanning global and conditional knockouts, knock-ins, and CRISPR/Cas9 editing, can separate molecular events that are necessary, sufficient, or merely correlated. These models show, for example, that enhanced trypsinogen autoactivation alone is sufficient for spontaneous disease, that NF-κB-driven inflammation can be initiated independently of trypsin, and that loss of autophagy alone disrupts acinar homeostasis. On this basis we reframe pancreatitis as a network pathophysiology with two coupled layers. First, acinar homeostasis is maintained by several semi-independent control modules (digestive-enzyme safety, endoplasmic reticulum protein quality control, the autophagy–lysosome system, the calcium–mitochondria axis, and NF-κB signaling), so that disease can be initiated at any of several independent nodes. Second, the injured acinar cell dies through an interconnected, switchable network of regulated cell death (apoptosis, necroptosis, pyroptosis, ferroptosis, and PANoptosis), sharing nodes such as caspase-8, RIPK3, and GPX4, whose configuration, more than the initiating trigger, determines severity. This framework recasts the heterogeneity of human pancreatitis as different entry points into one network and argues for therapies directed at shared nodes rather than single pathways.

## 1 Introduction

Pancreatitis remains a major cause of gastrointestinal morbidity worldwide. The Global Burden of Disease Study documented a rise from 1.73 million to 2.75 million cases between 1990 and 2021, imposing a growing burden on healthcare systems globally (Li et al., 2024). While most acute pancreatitis episodes are mild and self-limiting, approximately one-fifth of patients progress to severe acute pancreatitis characterized by persistent organ failure, with mortality reaching 20–40% when complicated by infected pancreatic necrosis (Boxhoorn et al., 2020; Párniczky et al., 2025). Beyond acute episodes, recurrent acute pancreatitis, chronic pancreatitis, and hereditary forms collectively reflect a clinical spectrum shaped by the interplay between intrinsic acinar vulnerability and extrinsic environmental insults. Despite this rising burden, no mechanism-specific therapy currently exists for pancreatitis, and clinical management remains largely supportive—a translational gap that ultimately traces back to incomplete mechanistic understanding at the cellular and molecular levels.

For over a century, pancreatitis pathogenesis was understood through the autodigestion hypothesis introduced by Chiari in 1896, which framed the disease as a single linear cascade initiated by premature intracellular trypsinogen activation, followed by sequential proteolytic activation of other zymogens and subsequent tissue destruction. This trypsin-centric framework gradually accumulated support from multiple converging lines of evidence over the twentieth century, including biochemical detection of activated digestive enzymes in pancreatitis specimens, reliable induction of pancreatitis phenotypes by diverse experimental insults such as supramaximal cerulein hyperstimulation, retrograde bile salt infusion, and L-arginine overload, and the genetic identification of the PRSS1 R122H mutation as a cause of hereditary pancreatitis. Yet this multi-source evidentiary base carries a fundamental methodological limitation. Chemical induction protocols and population genetic associations establish stimulus-level or population-level causal connections to pancreatitis but cannot resolve causality at the level of individual molecular events. Cerulein hyperstimulation, for instance, concurrently elicits intracellular trypsinogen activation, calcium signaling derangement, NF-κB pathway activation, endoplasmic reticulum stress (Saluja et al., 1997; Sah et al., 2013; Barrera et al., 2018; Luo et al., 2025); the model itself cannot distinguish which of these concurrent events is necessary for disease, which is sufficient, and which is a parallel bystander. Resolving such molecular-event-level causality requires experimental approaches that can isolate individual molecular events from their normal cellular context—the methodological capability that genetically engineered mouse models uniquely provide. The PACE-trypsinogen transgenic mouse developed by Ji and colleagues exemplifies this capability: by expressing a paired basic amino acid converting enzyme that directly cleaves trypsinogen within the secretory pathway, the model bypasses cathepsin B activation, and other upstream events typically engaged by chemical induction. The resulting selective activation of intracellular trypsin alone produced acinar injury and acute pancreatitis, establishing trypsin activity itself as sufficient to drive disease—a molecular-event-level sufficiency conclusion unattainable through chemical induction or population genetic association alone (Gaiser et al., 2011). The transition from chemical induction to GEMM-based investigation thus reflects not merely a technological update but a fundamental advance in the kind of causal questions that can be experimentally addressed.

Genetically engineered mouse models (GEMMs) provide the causal inference power that distinguishes necessary from sufficient from merely correlated molecular events. Loss-of-function studies using gene knockouts establish pathway necessity by determining whether a phenotype is abolished when a specific gene product is removed, while gain-of-function studies using knock-in or transgenic approaches establish sufficiency by determining whether introducing a defined molecular alteration is itself adequate to produce disease. The advent of CRISPR/Cas9-mediated genome editing has further expanded the GEMM toolkit, enabling precise modeling of human disease-causing variants at endogenous loci with unprecedented efficiency and flexibility. Across the past three decades, pancreatitis-related GEMMs have systematically dissected multiple mechanistic dimensions of disease pathogenesis. Knockouts of Ctsb and Spink1 (legacy name Spink3), together with transgenic expression of human PRSS1 R122H variants, have collectively elucidated digestive enzyme safety regulation, revealing how cathepsin-mediated activation, endogenous inhibitor function, and cooperative effects between mutant and wild-type trypsinogen isoforms govern intra-acinar protease homeostasis (Halangk et al., 2000; Ohmuraya et al., 2005; Huang et al., 2020; Wang J. et al., 2022). Pancreas-specific Atg5 and Atg7 conditional knockouts have established autophagy-lysosomal clearance as a core homeostatic function whose disruption alone is sufficient to drive spontaneous pancreatitis (Antonucci et al., 2015; Diakopoulos et al., 2015). Knock-in of misfolding-prone CPA1 variants has identified protein quality control as an independent pathogenic axis operating in parallel with trypsin-centered pathways (Hegyi and Sahin-Tóth, 2019). Slc7a11 and Gsdmd knockouts have most recently revealed that distinct regulated cell death programs—including ferroptosis and pyroptosis—shape the trajectory of injured acinar cells toward different clinical outcomes (Gao et al., 2021; Pan et al., 2023). Together, these GEMM-derived insights constitute the principal empirical foundation of modern pancreatitis mechanism research and make possible an integrative reading of pancreatitis pathogenesis that constitutes the central proposition of this review.

We propose that pancreatitis pathogenesis is best understood not as a single linear cascade but as a network pathophysiology process. Acinar homeostasis is maintained by several coupled functional modules, including digestive-enzyme safety, endoplasmic reticulum protein folding, autophagy-lysosomal and mitochondrial quality control, calcium signaling, and NF-κB and inflammasome-mediated inflammatory restraint. These modules are connected through shared molecular nodes, including autophagy, calcium dynamics, the cathepsin system, and mitochondrial function. Disruption of this network may arise from a dominant defect in one module, propagate across modules through shared coupling points, or occur through parallel perturbation of multiple modules by exogenous stress. GEMM studies illustrate each of these possibilities: accelerated trypsinogen autoactivation in T7D23A knock-in mice is sufficient to drive spontaneous pancreatitis (Geisz and Sahin-Tóth, 2018), whereas impaired autophagy-lysosomal clearance in *Atg7* conditional knockout mice is accompanied by broader disturbances in ER stress, mitochondrial function, and proteostasis (Antonucci et al., 2015). In chemically induced models such as cerulein hyperstimulation, intra-acinar trypsinogen activation, calcium dysregulation, NF-κB activation, and endoplasmic reticulum stress occur concurrently, illustrating how exogenous stress can engage several pathogenic modules in parallel. These network disturbances converge on a transition from acinar homeostasis to cellular injury. After this transition, disease severity is shaped in part by the regulated cell death program that is engaged. Apoptotic execution is generally associated with more contained injury, whereas necroptosis, pyroptosis, ferroptosis, and PANoptosis can release damage-associated molecular patterns and amplify inflammation. Plasticity and compensatory switching among these execution programs may further determine how an injured acinar cell reaches its terminal state. This network pathophysiology framework integrates the organization, collapse, and execution decisions of acinar homeostasis into a coherent conceptual architecture supported by GEMM evidence. It uses network-based mechanistic reasoning without requiring formal network-medicine methods such as disease-module analysis or network propagation.

This review examines the GEMM evidence supporting the network pathophysiology framework, traces its translational implications for stratified therapeutic strategies, and identifies open questions for future investigation.

## 2 GEMM as tools for dissecting the network pathophysiology of acinar cell homeostasis in pancreatitis

### 2.1 Digestive enzyme safety management: genetic dissection of trypsin activation, inhibition, and degradation

#### 2.1.1 The Chiari autodigestion hypothesis as the conceptual entry point for digestive enzyme GEMMs

The intellectual origin of modern pancreatitis research is conventionally traced to the autodigestion theory proposed by Hans Chiari in 1896. Based on his observation of autolytic phenomena in autopsied pancreata, Chiari proposed that pancreatitis is a disease caused by aberrant intrapancreatic activation of digestive enzymes. This hypothesis offered a critical testable proposition for subsequent research: if pancreatitis is indeed driven by aberrant intra-acinar activation of digestive enzymes, then precise genetic manipulation targeting digestive enzyme genes or their regulatory pathways should recapitulate pancreatitis phenotypes in mice.

The autodigestion hypothesis predicted three classes of molecularly tractable events: increased aberrant activation of trypsinogen, insufficient inhibition of generated trypsin, and failure to degrade or inactivate trypsinogen and trypsin. These three classes correspond to three categories of core GEMMs that have subsequently been developed: models targeting trypsinogen activation control (encompassing both trypsinogen autoactivation variants and knockout of the lysosomal activating enzyme CTSB); models targeting trypsin degradation failure (encompassing knockout of the lysosomal degrading enzyme CTSL together with knockout of the secretory degrading enzymes CTRC and CTRB1); and models targeting the endogenous inhibitor SPINK1/Spink3.

#### 2.1.2 PRSS1 transgenic mice: from human genetic discovery to first-generation disease relevant GEMMs

The experimental foundation of the trypsin-centric framework traces back to chemically induced pancreatitis models developed between the 1970s and 1990s, including supramaximal cerulein stimulation, retrograde taurocholate infusion into the pancreatic duct, high-dose L-arginine administration, and the choline-deficient ethionine-supplemented (CDE) diet (Lampel and Kern, 1977; Lerch and Gorelick, 2013).These models had already accumulated substantial evidence supporting intra-acinar trypsinogen activation as a central early event in pancreatitis, establishing the trypsin-centric framework as the dominant paradigm of mechanistic research. However, these chemical models could only recapitulate pancreatitis phenotypes under experimental conditions and could not address a more fundamental question: whether intrinsic abnormalities in digestive enzyme genes themselves can directly drive disease in spontaneous or hereditary settings.

Through linkage analysis of hereditary pancreatitis (HP) families, Whitcomb and colleagues identified the R122H missense mutation in PRSS1 as the genetic basis of autosomal dominant hereditary pancreatitis (Whitcomb et al., 1996). R122H is located at the autolytic cleavage site of active trypsin, and its pathogenic mechanism is to eliminate this site, thereby protecting active trypsin from endogenous degradation and elevating aberrant pancreatic trypsin activity. The historical significance of this discovery lies in providing the first direct human genetic evidence for a trypsin-centric framework that had already dominated the field, transforming Chiari’s autodigestion theory from a clinical hypothesis grounded in autopsy observations into a precise mechanistic proposition with human genetic support.

R122H is the prototypical but by no means the only PRSS1 variant that causes classic autosomal-dominant hereditary pancreatitis (Whitcomb et al., 1996). A broader allelic series has since been identified in hereditary-pancreatitis cohorts, including A16V (Witt et al., 1999), N29I (Gorry et al., 1997), and N29T and R122C (Pfützer et al., 2002), among others—and functional dissection of these human cationic trypsinogen variants shows that they do not act through a single lesion but through three mechanistically distinct routes that converge on acinar injury (**Table 1**). The first two routes both elevate intra-acinar trypsin activity. Some variants enhance trypsinogen autoactivation, accelerating the conversion of trypsinogen to active trypsin (e.g., A16V, N29I). Others impair the protective degradation of trypsinogen and trypsin—most directly by eliminating the Arg122 autolysis site through which active trypsin normally self-degrades (R122H, R122C), or by rendering the zymogen more resistant to proteolytic breakdown (N29T, V39A). A third route is independent of trypsin activity: misfolding variants (R116C (Kereszturi et al., 2009), C139F (Schnúr et al., 2014)) disrupt the folding and secretion of trypsinogen, triggering endoplasmic-reticulum stress and the unfolded-protein response—the same mechanism captured by the T7C140S mouse in Section 2.1.3 and developed further in Section 2.4. This demonstrates that the rise in intra-acinar trypsin activity central to the autodigestion hypothesis is only one of several molecular routes to hereditary pancreatitis, and that a single gene—PRSS1—can drive disease through convergent yet mechanistically independent failure modes. This convergence foreshadows the network logic developed throughout this review and explains why the murine modeling effort described below has treated R122H as a representative rather than an exclusive disease allele.

**Table 1:** Molecular mechanisms of hereditary pancreatitis–associated human cationic trypsinogen variants.

Mechanistic class	PRSS1 variant	Dominant molecular mechanism	Consequence for the acinar cell	Ref. (mechanism)
Enhanced autoactivation	A16V	Accelerates removal of the activation peptide, speeding conversion of trypsinogen to active trypsin	autoactivation	Nemoda and Sahin-Tóth, 2006
	N29I	Enhances trypsinogen autoactivation, with added resistance to degradation	autoactivation	Szabó and Sahin-Tóth, 2012
Impaired protective degradation	N29T	Increases resistance to proteolytic degradation of trypsinogen and trypsin	degradation	Szabó and Sahin-Tóth, 2012
	V39A	Increases resistance to proteolytic degradation of trypsinogen and trypsin	degradation	Szabó and Sahin-Tóth, 2012
	R122C	Eliminates the Arg122 autolysis site, blocking self-degradation of active trypsin; unpaired Cys can also promote misfolding	degradation (± misfolding)	Simon et al., 2002; Szabó and Sahin-Tóth, 2012
	R122H	Eliminates the primary Arg122 autolysis site, protecting active trypsin from self-degradation	degradation	Whitcomb et al., 1996; Szabó and Sahin-Tóth, 2012
Misfolding/ER stress (trypsin-independent)	R116C	Mutation-induced misfolding with intracellular retention and aggregation	ER stress; secretion defect	Kereszturi et al., 2009
	C139F	Misfolding with a severe secretion defect	ER stress; secretion defect	Schnúr et al., 2014

Modeling human PRSS1 R122H in mice has followed a convoluted trajectory. Selig et al. first expressed human PRSS1 R122H in mice through random integration transgenesis driven by an elastase promoter, but transgene expression was too low to yield an overt pancreatitis phenotype (Selig et al., 2006). Athwal et al., using the rat elastase promoter, found that expression of WT or mutant human PRSS1 in murine acinar cells produced a spontaneous pancreatitis-like phenotype (acinar vacuolisation, inflammatory infiltrates, fibrosis) accompanied by increased acinar apoptosis, with further worsening upon cerulein challenge (Athwal et al., 2014). Huang et al. employed a full-length elastase promoter-driven PRSS1 R122H transgene and demonstrated markedly increased susceptibility to pancreatitis induced cerulein, LPS, or alcohol, although basal pancreatic trypsin activity was not elevated and no spontaneous pancreatitis developed (Huang et al., 2020). Gui by et al. introduced an enzymatically dead control variant (PRSS1R122H, S200T) to show that this sensitizing effect requires trypsin enzymatic activity (Gui et al., 2019). Wan et al. demonstrated through transgenic expression of human PRSS2 alone that wild-type human PRSS2 can also aggravate murine pancreatitis (Wan et al., 2020). Wang et al. observed a spontaneous hereditary pancreatitis-like phenotype only when PRSS1 R122H and wild-type PRSS2 were co-expressed in a compound transgenic strategy (Wang J. et al., 2022), revealing cooperative pathogenic effects between different trypsinogen isoforms.

All of these mouse models expressing human PRSS1 R122H protein have uniformly relied on random-integration transgenesis, a methodological necessity rooted in the absence of a true homologous sequence for human PRSS1 in the mouse genome (the cationic trypsinogen isoform in mice is T7). The fundamental nature of random-integration transgenesis—the insertion of exogenous DNA constructs at random genomic loci—introduces several unavoidable sources of artifact: position effects from the chromatin environment at the insertion site, copy-number dependence from multi-copy tandem integration, non-physiological spatiotemporal expression driven by exogenous promoters, and potential disruption of endogenous gene function at the insertion site. These methodological limitations collectively introduce systemic imprecision into genotype–phenotype correlations and laid the groundwork for the subsequent T7-locus knock-in models based on precision endogenous editing.

#### 2.1.3 T7 trypsinogen models: precision endogenous editing reveals the spectrum of trypsin GEMMs

A major methodological advance in trypsinogen GEMMs came from endogenous-locus engineering of the mouse T7 trypsinogen gene, the major cationic trypsinogen isoform in the mouse pancreas. This strategy allows mutant trypsinogen to be expressed at physiological levels and in physiological spatiotemporal patterns, thereby eliminating the overexpression artifacts, position effects, and insertional mutagenesis intrinsic to random integration transgenesis.

The loss-of-function arm of the T7 series is exemplified by the global T7-/- knockout mouse generated by Dawra and colleagues through homologous recombination (Dawra et al., 2011), the first GEMM in the digestive enzyme safety management field directly targeting trypsinogen itself. In cerulein-induced acute pancreatitis, T7-/- mice exhibit nearly complete loss of intra-acinar trypsin activity and markedly attenuated early acinar injury, yet inflammatory responses (NF-κB activation, cytokine release, and inflammatory cell infiltration) remain comparable to those in wild-type animals. This genetic evidence directly challenged the central assumption of the trypsin-centric framework and redefined the role of trypsin as an important mediator of early acinar injury but not the master switch of the broader inflammatory cascade. Sah and colleagues extended this finding to chronic disease by demonstrating that cerulein-induced chronic pancreatitis likewise does not require intra-acinar trypsinogen activation (Sah et al., 2013). The T7-/- model thus provides the key loss-of-function experimental foundation for the independent pathogenic role of inflammatory pathways discussed in Section 2.2.

The autoactivation-enhancement arm is exemplified by the T7D23A knock-in mouse generated by Geisz and Sahin-Tóth (2018), an engineered gain-of-function variant rationally designed from biochemical principle rather than copied from any human pathogenic mutation. The D23A substitution accelerates trypsinogen autoactivation approximately 50-fold, and even heterozygous *T7D23A* mice spontaneously develop acute pancreatitis that progresses to chronic disease—with acinar loss, fibrosis, and mononuclear infiltration—in the complete absence of any exogenous trigger. This established the central sufficiency claim: enhanced endogenous autoactivation alone can drive the full acute-to-chronic continuum. Whether autoactivation is invariably sufficient, however, is answered by the companion *T7K24R* knock-in, which models human PRSS1 K23R and raises autoactivation only ~5-fold (Jancsó and Sahin-Tóth, 2020). *T7K24R* mice develop no spontaneous disease, yet they sustain markedly more severe cerulein-induced acute pancreatitis than controls—and, tellingly, a single cerulein episode is enough to commit them to chronic pancreatitis (acinar atrophy, persistent macrophage infiltration, diffuse fibrosis) long after the initiating injury has ceased, whereas wild-type mice recover fully (Jancsó and Sahin-Tóth, 2022). Reasoning that the contrasting phenotypes of the 50-fold *T7D23A* and 5-fold *T7K24R* strains implicated the rate of autoactivation as the key determinant of disease, Demcsák and Sahin-Tóth tested this directly by engineering an intermediate variant, *T7D22N,K24R*, whose autoactivation is accelerated ~13-fold (Demcsák and Sahin-Tóth, 2022); homozygotes develop spontaneous disease, but with later onset and slower progression than *T7D23A* mice. The three strains together trace a monotonic rate–phenotype relationship (~5-, ~13-, and ~50-fold), which we read as evidence that spontaneous pancreatitis is governed by a rate-dependent threshold rather than by the presence of autoactivation per se—reframing trypsin not as a binary switch at the head of a linear cascade, but as a tunable node whose output, once past threshold, can ignite self-perpetuating downstream inflammation.

The disease-orthologous knock-in arm is exemplified by the T7R123H mouse generated by Jancsó and colleagues (Jancsó et al., 2023b), which installs the precise mouse equivalent of human PRSS1 R122H at the corresponding T7 position. Homozygous T7R123H mice develop no spontaneous pancreatitis, and short course cerulein produces severity only marginally above C57BL/6N controls; only under sustained cerulein (two consecutive days) does a partial phenotype emerge, with roughly 40% of T7R123H mice progressing to atrophic pancreatitis while controls recover fully. This graded result is more informative than a flat null: it shows that the mouse counterpart of R122H is active but insufficient—capable of modestly amplifying injury, yet unable to initiate spontaneous disease at physiological expression. Mechanistically, the mutation only inefficiently blocks CTRB1 (chymotrypsin B1)–dependent degradation of mouse trypsinogen, which both explains its weak effect and identifies the relevant context: the pathogenicity of human R122H depends on its ability to defuse chymotrypsin-C–mediated defenses, a dependence the mouse enzyme does not fully reproduce. Because precision endogenous knock-in had removed the copy-number, position, and overexpression artifacts that confounded the transgenic models in Section 2.1.2, the negative result in T7R123H mice carries weight that those earlier models could not: at physiological expression, the mouse counterpart of R122H does not drive spontaneous pancreatitis. The T7C140S knock-in mouse recently developed by Sándor and colleagues extends the T7 series from trypsinogen activation mechanisms to protein misfolding mechanisms: the T7C140S mutation removes a cysteine required for correct disulfide bonding, leading to protein misfolding and endoplasmic reticulum accumulation, and homozygous T7C140S mice develop chronic pancreatitis with ER stress and unfolded protein response (UPR) activation (Sándor et al., 2025). This establishes that misfolding of trypsinogen itself, independent of any change in its activation, is sufficient to drive disease, and it provides a bridging case to the protein-quality-control pathway of Section 2.4. The broader lesson spans the whole series: a single gene—cationic trypsinogen—can drive pancreatitis through two mechanistically independent routes, aberrant activation and aberrant folding, underscoring why pancreatitis is better read as a network of convergent failure modes than as a single linear cascade.

Compound genotypes combining *T7* mutants with other members of the digestive-enzyme-safety pathway expose within-pathway, multi-gene synergy and recapitulate the multi-variant coexistence common in human pancreatitis (Jancsó et al., 2023a). The *Spink1*-KO^het^ × *T7D23A* cross—pairing loss of trypsin-inhibitor capacity with enhanced autoactivation—produces a markedly aggravated phenotype (Demcsák and Sahin-Tóth, 2024), and the *T7C140S* × *Cpa1* N256K cross—combining two independently misfolding digestive enzymes—causes more severe pancreatic atrophy and ER stress than either single-gene model (Sándor et al., 2025). These designs achieve precise correspondence between GEMM genotype and the combinatorial genetic risk seen in patients.

The T7 series collectively exposes one common methodological limitation—the complexity of the mouse trypsinogen gene family. The mouse genome contains approximately 20 trypsinogen-related genes; four isoforms are expressed at high levels in the resting pancreas (T7, T8, T9, and T20), with T7 accounting for only 40–50% of total trypsinogen (Németh et al., 2013). Single-gene knockout or editing of T7 cannot fully eliminate the contribution of endogenous trypsinogen, as T8, T9, and T20 together still account for 50–60% of total mouse pancreatic trypsinogen output. Ji and colleagues addressed this constraint through a streamlined genotyping workflow for CRISPR/Cas9-edited mice, using the mouse trypsinogen gene family as a representative application to demonstrate the feasibility of simultaneously editing multiple trypsinogen isoform genes (Wang et al., 2024). This advance represents a methodological progression from single-gene precision editing toward family-level editing of the entire trypsinogen repertoire, and the resulting family-wide knockout models will ultimately be able to address a long-standing open question within the trypsin-centric framework: whether pancreatitis can still occur in mice with complete elimination of intra-acinar trypsinogen expression.

#### 2.1.4 Lysosomal cathepsin GEMMs: *Ctsb*-mediated activation versus *Ctsl*-mediated degradation in the same compartment

Sections 2.1.2 and 2.1.3 addressed how mutations in trypsinogen itself determine trypsin activity. A more fundamental mechanistic question remains—through what enzymatic machinery is trypsinogen aberrantly activated within acinar cells? Halangk and colleagues answered this question through the global *Ctsb*^−^/^−^ knockout mouse (Halangk et al., 2000). In cerulein-induced acute pancreatitis, *Ctsb*^−^/^−^ mice show near-complete suppression of intrapancreatic trypsin activity and markedly attenuated acinar necrosis, establishing the lysosomal cysteine protease CTSB as the principal executor of aberrant intra-acinar trypsinogen activation. This mechanism rests on the long-standing colocalization hypothesis: pancreatitis-inducing insults disrupt the normal intracellular segregation of lysosomal hydrolases from digestive zymogens, driving their pathological colocalization within cytoplasmic compartments where CTSB can cleave and activate trypsinogen (Saluja et al., 2019).

The global model carries an interpretive limitation, however: CTSB is expressed not only in acinar cells but also in macrophages and other immune cells, so whole-body deletion cannot resolve which cell type’s CTSB drives disease. Two studies sharpened this picture from opposite directions. Sendler and colleagues showed, ex vivo and *in vivo*, that pancreas-infiltrating macrophages phagocytose acinar-derived zymogen granules and activate trypsinogen via macrophage-intrinsic CTSB; crucially, this intra-macrophage activation triggers NF-κB translocation and pro-inflammatory cytokine release, so it does not merely add a second source of trypsin but actively amplifies systemic inflammation and disease severity—indeed, transfer of wild-type macrophages into *Ctsb*^−^/^−^ recipients was sufficient to worsen pancreatitis (Sendler et al., 2018). Chen and colleagues, conversely, used Cre-loxP–mediated pancreas-specific deletion of *Ctsb* and *Ctsl* and found that this deletion significantly altered intrapancreatic trypsin activity yet had limited impact on acute pancreatitis severity (Chen et al., 2022), suggesting that CTSB-mediated trypsin activation is not the sole determinant of pancreatitis severity.

Within the same lysosomal compartment where CTSB activates trypsinogen, cathepsin L (CTSL) plays the opposite role. Wartmann and colleagues demonstrated biochemically that CTSL acts on trypsinogen at a different cleavage site than CTSB, producing an extended N-terminal oligopeptide and a truncated, enzymatically inactive trypsin (Wartmann et al., 2010). In other words, CTSB cleaves the trypsinogen activation peptide (TAP) to generate enzymatically active trypsin, whereas CTSL cleaves trypsinogen at a different site to generate an enzymatically inactive truncated fragment—two cleavage patterns with diametrically opposite functional consequences. CTSB serves as the trypsin-activating protease while CTSL serves as the trypsin-degrading protease. Through global *Ctsl*^−^/^−^ knockout mice, Wartmann et al. found that CTSL deficiency markedly increased intra-acinar TAP levels and trypsin activity in cerulein-induced pancreatitis, consistent with the biochemical prediction of CTSL as a trypsin-degrading enzyme. Paradoxically, however, *Ctsl*^−^/^−^ mice exhibited substantially reduced pancreatitis severity compared to wild-type mice. The resolution of this paradox lies in CTSL deficiency shifting acinar cell death from necrosis (highly inflammatory) toward apoptosis (relatively less inflammatory), thereby limiting overall disease severity. This result carries a deeper conceptual implication: elevated intra-acinar trypsin activity in mouse GEMMs does not necessarily produce more severe pancreatitis. The *Ctsl*^−^/^−^ finding is one of the key pieces of evidence supporting the subsequent reassessment of the true pathogenic role of trypsin in pancreatitis, and it also provides an early clue for the discussion in Section 2.5 of cell death execution programs as the determinants of acinar injury outcome.

#### 2.1.5 SPINK1/Spink3 models: limitations of conventional knockout strategies and the breakthrough of CRISPR-based precision editing

Witt and colleagues identified mutations in the SPINK1 gene, particularly N34S, as a genetic risk factor in patients with chronic pancreatitis (Witt et al., 2000). SPINK1 encodes the pancreatic secretory Kazal-type trypsin inhibitor, which neutralizes up to ~20% of potentially available trypsin activity (Zadrozny Gouvêa da Costa et al., 2011). The association of SPINK1 mutations with pancreatitis extended the research dimension of digestive enzyme safety management from excessive activation to insufficient inhibition following activation.

The genotype–phenotype relationship of SPINK1 N34S exhibits noteworthy complexity. Structural and biophysical analysis by Nagel and colleagues showed that the N34S mutation neither impairs SPINK1’s capacity to inhibit human cationic trypsin nor alters its interaction interface with trypsin (Nagel et al., 2022). The frequency of N34S in HP families is comparable to that in the general population, and linkage between pancreatitis and the SPINK1 locus has been excluded in hereditary-pancreatitis families (Threadgold et al., 2002). Current evidence indicates that the pathogenic signal within the N34S-containing haplotype is most plausibly explained not by the N34S substitution itself but by the co-inherited enhancer variant c.-4141G>T, which disrupts a PTF1L-binding site within a conserved HNF1A–PTF1L cis-regulatory module upstream of *SPINK1* and reduces acinar *SPINK1* transcription (Pu et al., 2021); the alternative, misfolding-driven route of intracellular retention and degradation applies to rarer missense variants such as D50E, Y54H, and R67C, but not to N34S (Kiraly et al., 2007). This distinction has a defining consequence for model design. Because the pathogenic determinant of N34S is a quantitative deficit in functional inhibitor rather than a qualitative defect in the protein, the appropriate genetic surrogate is not a mutation engineered into the coding sequence but a controlled reduction in inhibitor gene dosage—precisely the configuration that a heterozygous loss-of-function allele provides. What had remained technically out of reach, however, was the means to build such a model in the adult pancreas—a gap defined less by conceptual uncertainty than by the limitations of the available genetic tools.

For nearly two decades, GEMM-based interrogation of inhibitor function *in vivo* was constrained by the phenotypes of the available models. The murine orthologue is *Spink1* (historically designated *Spink3*). Global *Spink1*-deficient mice, generated by Ohmuraya and colleagues through homologous recombination, undergo autophagic death of pancreatic acinar cells beginning in late gestation and do not survive beyond approximately two weeks of age, with only limited inflammatory infiltration (Ohmuraya et al., 2005), precluding the use of this model for adult pancreatitis studies. Nathan and colleagues showed that transgenic overexpression of pancreatic secretory trypsin inhibitor (a rat SPINK1 paralogue) elevated endogenous inhibitory capacity and significantly attenuated cerulein-induced acute pancreatitis (Nathan et al., 2005). An adult model reproducing the heterozygous loss-of-function state characteristic of human *SPINK1* carriers, however, remained unavailable.

The advent of CRISPR/Cas9 gene editing fundamentally broke this methodological impasse. Demcsák and Sahin-Tóth used CRISPR/Cas9 to introduce precise editing directly at the mouse Spink1 ortholog locus, generating *Spink1*-KO^het^ heterozygous knockout mice (Demcsák and Sahin-Tóth, 2024). This design avoided the neonatal lethality associated with complete inhibitor loss and enabled analysis of partial SPINK1 deficiency in adult mice— the configuration that approximates the reduced inhibitor levels caused by heterozygous loss-of-function SPINK1 variants in human chronic pancreatitis. In this model, heterozygous *Spink1* deletion did not alter the response to limited cerulein hyperstimulation, but prolonged cerulein stimulation increased intrapancreatic trypsin activity, aggravated acute pancreatitis, and promoted progression to chronic pancreatitis-like disease. When introduced into trypsinogen autoactivation mutant backgrounds, including *T7D23A* and *T7D22N, K24R* mice, the *Spink1*-KO^het^ allele further exacerbated chronic pancreatitis. The study thus provides genetic evidence that partial SPINK1 deficiency increases pancreatitis susceptibility, an effect amplified in a trypsin-dependent manner when combined with enhanced trypsinogen autoactivation. Methodologically, it illustrates the value of endogenous-locus editing for modeling partial loss of function, bypassing homozygous lethality, and building compound-genotype models that more closely approximate the multigenic architecture of human pancreatitis.

#### 2.1.6 CTRC and CTRB1 models: the post-secretion trypsin degradation defense layer and strain-specific methodological pitfalls

Beyond the lysosomal compartment, the principal degradative defense against trypsin is mediated by secreted chymotrypsin. Rosendahl and colleagues established the association between *CTRC* (chymotrypsin C) variants and chronic pancreatitis (Rosendahl et al., 2008). Biochemical studies from the Sahin-Tóth laboratory elucidated CTRC’s core function: CTRC acts synergistically with trypsin to degrade trypsinogen, in which trypsin first converts chymotrypsinogen C into active CTRC, and CTRC then acts together with trypsin to fully degrade trypsinogen (Szmola and Sahin-Tóth, 2007). Together with the intra-lysosomal CTSL discussed in Section 2.1.4, CTRC constitutes the two spatial dimensions of trypsin degradation defense: CTSL degrades aberrantly activated trypsin within the lysosomal compartment, while CTRC degrades prematurely activated trypsin in the acinar secretory pathway.

CTRC GEMM research has revealed a methodological fact with broad implications for all murine pancreatitis modeling work. The widely used C57BL/6 strain does not express functional CTRC, owing to a natural single-nucleotide deletion (c.117delT) in exon 2 of the *Ctrc* gene that abolishes CTRC activity (Geisz et al., 2019). Anti-trypsin chymotryptic defense in the mouse is instead carried predominantly by CTRB1 (chymotrypsin B1), which accounts for roughly 90% of total pancreatic chymotrypsin—an arrangement common to laboratory mice generally, rather than a compensatory response to the C57BL/6 *Ctrc* defect. Restoring a functional *Ctrc* locus in C57BL/6 mice significantly attenuated cerulein-induced trypsinogen activation, both confirming a protective role for CTRC *in vivo* and illustrating a broader methodological principle: the genetic background of a mouse strain is itself an experimental variable, one capable of silently shaping the interpretation of pancreatitis phenotypes. Consistent with the dominant role of CTRB1, deletion of *Ctrb1* (*Ctrb1*-del) aggravated cerulein-induced acute pancreatitis (Jancsó et al., 2018), and combining *Ctrb1*-del with an autoactivating trypsinogen mutant background yielded spontaneous chronic pancreatitis (Jancsó et al., 2023a). Such compound-genotype studies establish the centrality of the degradative defense layer and again demonstrate within-pathway, multi-gene synergy: a single-gene defect may be insufficient to precipitate disease, whereas concurrent defects across several genes can breach the integrated defense network—reproducing, in the mouse, the combinatorial genetic architecture characteristic of human pancreatitis.

### 2.2 Acinar inflammatory response: the NF-κB pathway as a pathogenic module

#### 2.2.1 From downstream executor of trypsin to independent initiator of inflammation

Under the classical trypsin-centric framework, inflammation in pancreatitis was traditionally understood as a downstream consequence of aberrant trypsin activation and acinar cell injury. Studies in the early 2000s, however, gradually reshaped this understanding. Gukovsky and colleagues showed that NF-κB is activated early during hormone-induced experimental pancreatitis, identifying inflammatory transcriptional signaling as one of the earliest acinar responses to pathological stimulation (Gukovsky et al., 1998). This finding raised an important mechanistic question: was NF-κB activation merely downstream of trypsinogen activation, or could it be engaged as an independent pathogenic program?

Han and colleagues addressed this question in pancreatic acinar cells. In CHO-CCK(A) cells, which express the CCK(A) receptor but lack trypsinogen, CCK activated NF-κB in the absence of trypsinogen; conversely, adenoviral activation or inhibition of NF-κB altered neither basal nor CCK-mediated trypsinogen activation (Han et al., 2001). Hietaranta and colleagues reported consistent findings in a separate system: lipopolysaccharide and phorbol ester induced NF-κB activation without trypsinogen activation, while the chymotrypsin inhibitor TPCK suppressed NF-κB activation without affecting trypsin activity (Hietaranta et al., 2001). These results indicate that NF-κB and trypsinogen activation are temporally coupled but separately triggered, with neither strictly required for the other.

This distinction changed how the acinar cell was conceptualized in pancreatitis. Rather than serving only as a passive target of digestive enzyme-mediated injury, the acinar cell can actively initiate inflammatory transcriptional programs. Early inflammatory signaling therefore does not necessarily require preceding trypsin-mediated damage; pathological stimuli can directly engage the acinar NF-κB pathway and drive inflammatory gene expression.

#### 2.2.2 *In vivo* and GEMM evidence for the pathogenic sufficiency of NF-κB

The *in vitro* decoupling evidence summarized above, while compelling, remained at the level of indirect cellular observations. Whether the NF-κB pathway could independently induce pancreatitis at the whole-organism level required direct testing through genetically engineered mouse models. Chen and colleagues provided the first *in vivo* gain-of-function evidence for the sufficiency of NF-κB activation: intraductal delivery of an adenoviral vector encoding constitutively active p65 (Adp65) induced acinar injury, neutrophil infiltration, and secondary pulmonary inflammation, whereas co-delivery of inhibitory IκBα substantially attenuated this phenotype (Chen et al., 2002). These results support that acinar NF-κB activation alone can induce pancreatitis-characteristic injury without requiring trypsin activation as a prerequisite. Baumann et al. complemented this evidence through acinar cell-specific *Ikk2*-regulated mice based on the rtTA-tetO system. IKK2-CA mice developed typical pancreatitis features after doxycycline induction; complementarily, IKK2-DN mice showed attenuated phenotypes under cerulein challenge while cerulein-induced trypsin activation remained unaffected—further supporting the relative independence between the NF-κB pathway and trypsin activation at the acinar cell-specific level (Baumann et al., 2007).

The companion paper by Algül et al. revealed that NF-κB plays far more than a single “pro-inflammatory pathogenic” role. In acinar cell-specific *RelA/p65* truncation mice, RelA deficiency did not attenuate but rather aggravated cerulein-induced acinar injury and systemic complications; mechanistically, RelA in acinar cells drove the expression of the protective acute-phase protein PAP1, protecting acinar cells from necrosis (Algül et al., 2007). This finding suggests that NF-κB in acinar cells may possess a dual role—driving inflammatory pathogenesis when excessively activated, while maintaining acinar homeostasis through induction of protective proteins under physiological activation. This duality will be further elucidated in Section 2.2.4.

#### 2.2.3 Bidirectional decoupling of NF-κB and trypsin activation

Chen et al. (2002) and Baumann et al. (2007) supported the relative independence of the acinar inflammatory module from the direction of “NF-κB activation is sufficient for pathogenesis.” Evidence from the opposite direction—”loss of trypsin but preserved inflammatory response”—came from the T7 series knockout mice discussed in Section 2.1.3. Dawra et al. observed a notable phenomenon in *T7*^-/-^ whole-body knockout mice during acute pancreatitis—cerulein-induced intra-acinar trypsin activation was almost completely abolished, yet NF-κB activation and tissue inflammatory response remained comparable to wild type; early markers of acinar injury were reduced by approximately 50%, but the core features of inflammatory response were unaffected (Dawra et al., 2011). Sah et al. extended this finding to the chronic phase—repeated cerulein-induced chronic pancreatitis in *T7^-/-^* and *Ctsb^-^*^/-^ mice still produced fibrosis, acinar loss, and inflammatory cell infiltration similar to wild type (Sah et al., 2013). Ji et al. (2009) provided “reverse decoupling” evidence—trypsin activated but NF-κB not activated. Using a PACE-trypsinogen mutant to achieve pure intra-acinar trypsin activation without exogenous stress, intracellular trypsin activation induced apoptosis in acinar cells but had no significant effect on NF-κB activity; extracellular trypsin, by contrast, induced both cellular injury and NF-κB activation. This work, together with the T7*^-/-^* studies, jointly supports the decoupling of the two events.

At this point, the relative independence between the acinar inflammatory module and the digestive enzyme safety management module has gained support from multiple layers of evidence—*in vitro* decoupling, GEMM evidence for pathogenic sufficiency, and bidirectional GEMM decoupling. This finding is one of the important empirical foundations of the network pathophysiology framework—it suggests that acinar homeostatic collapse can be initiated through two distinct entry points rather than along a single linear cascade.

#### 2.2.4 Refined dissection of the dual role of the NF-κB pathway

Once GEMM data established the NF-κB pathway as a pathogenic module, attention turned to a more specific question: in acinar cells, does NF-κB act mainly as a pro-inflammatory driver or as a protective factor? A pair of companion papers, published together with an accompanying editorial, indicated that the answer is conditional rather than categorical.

Huang et al. used Ela-CreERT × LSL-Ikk2 double-transgenic mice to drive tamoxifen-inducible, sustained NF-κB activation in acinar cells. Short-term activation reproduced features of acute pancreatitis, whereas sustained activation over three months produced features of chronic pancreatitis, including acinar loss, interstitial fibrosis, and stellate-cell activation (Huang et al., 2013). Sustained acinar NF-κB activation is therefore sufficient, on its own, to drive a chronic-pancreatitis phenotype. The companion study by Neuhöfer et al. took the opposite approach, deleting *Ikba* in the pancreas via *Ptf1a-cre × Ikba^F/F^*. Contrary to the expectation that loss of the principal NF-κB inhibitor would aggravate disease, *Ikba*-deficient mice showed attenuated acute pancreatitis; constitutive nuclear RelA up-regulated the protective target gene *Spi2a*, and lentiviral Spi2A expression was itself protective (Neuhöfer et al., 2013). This result points to a second role for the pathway: sustaining acinar resistance to injury through protective target genes. In an accompanying editorial, Gukovsky and Gukovskaya placed both findings within a single framework. Because the NF-κB regulon spans both pro-inflammatory mediators and cytoprotective proteins, the net effect of activation depends on its intensity and duration, the composition of the active complexes, and the other pathways engaged in parallel (Gukovsky and Gukovskaya, 2013). This framework helps explain the apparent paradox of the NF-κB pathway and illustrates the tunability of acinar homeostatic regulation.

Chan et al. showed the complexity of NF-κB roles from another dimension through acinar cell-specific NEMO (IKKγ) knockout mice. *NEMO*^Δpan^ mice exhibited aggravated inflammation, fibrosis, and acinar-to-ductal metaplasia in the repeated cerulein-induced chronic pancreatitis model, with mechanisms involving sustained activation of the CXCL12/CXCR4 axis (Chan et al., 2017). This work suggests that the NF-κB pathway may possess differential roles across different disease phases and different downstream mediators. Li et al. revealed a notable phenomenon through acinar cell-specific IKKα knockout mice—IKK complex components may regulate acinar homeostasis through mechanisms not dependent on NF-κB. *Ikkα^Δpan^* mice spontaneously developed a phenotype resembling human chronic pancreatitis, with mechanisms involving defective autophagic protein degradation, p62-mediated protein aggregate accumulation, and ER stress (Li et al., 2013). This work repositioned IKKα as a regulator participating in autophagic protein degradation, creating a conceptual bridge between the inflammatory signaling pathway (Section 2.2) and the autophagy-lysosome pathway (Section 2.3).

Taken together, the genetically engineered mouse models discussed in this section, summarized in **Table 2**, demonstrate that NF-κB is neither inherently pathogenic nor inherently protective. Instead, it functions as a context-dependent regulator of acinar homeostasis, with the outcome of activation determined by its duration and magnitude (transient versus sustained IKK2/NF-κB activation), the specific downstream target genes engaged (pro-inflammatory mediators versus cytoprotective proteins such as PAP1 and Spi2a), and its interactions with parallel pathways—including chemokine signaling (the CXCL12/CXCR4 axis in NEMO-deficient mice) and autophagy (the NF-κB–independent role of IKKα in autophagic protein degradation). This context dependence reconciles the apparently paradoxical phenotypes described above and reframes the NF-κB module not as a unidirectional pathogenic switch but as a tunable node within the acinar homeostatic network.

**Table 2:** Genetically engineered mouse models dissecting the dual, context-dependent role of NF-κB in the exocrine pancreas.

Mouse model	NF-κB status	Phenotype	Conclusion	Ref.
IKK2-CA	Constitutive activation	Acute progressing to chronic pancreatitis (acinar loss, fibrosis, stellate-cell activation)	Sustained NF-κB activation is, on its own, sufficient to drive disease (pathogenic)	Huang et al., 2013
IκBα knockout	Constitutive nuclear RelA	Attenuated acute pancreatitis	NF-κB induces protective target genes (e.g., Spi2a)	Neuhöfer et al., 2013
NEMO (IKKγ) knockout	Canonical NF-κB impaired	Aggravated chronic pancreatitis	Basal NF-κB maintains homeostasis; its loss enables CXCL12/CXCR4-driven injury	Chan et al., 2017
IKKα knockout	NF-κB–independent defect	Spontaneous chronic pancreatitis	IKKα sustains homeostasis via autophagic protein degradation, independent of NF-κB	Li et al., 2013

### 2.3 Organelle homeostasis maintenance: the autophagy–lysosome system from trypsin activation compartment to acinar homeostasis core

#### 2.3.1 Early GEMM observations and the trypsin activation compartment hypothesis

The autophagy–lysosome system is the principal quality-control network of the acinar cell, responsible for degrading misfolded proteins, damaged organelles, and zymogen granules that are not secreted in a timely manner. Its conceptual role in pancreatitis, however, has not been static: the field’s initial reading of autophagy as a pathogenic contributor stands in contrast to the interpretation that later GEMM evidence would support.

Autophagy research initially remained embedded within the trypsin-centric framework, with the core question being—in which subcellular compartment does trypsinogen become aberrantly activated within acinar cells? Around this question, the work of Hashimoto et al. can be regarded as an early entry point. In acinar-cell–specific *Atg5*-knockout mice (*Atg5^flox/flox^;EL-Cre2*), cerulein-induced acute pancreatitis was markedly attenuated and intra-acinar trypsinogen activation was greatly reduced (Hashimoto et al., 2008). Based on this phenotype, the authors proposed the “autophagy hypothesis of trypsinogen activation”—autophagy delivers trypsinogen to the acidic environment containing lysosomal hydrolases (such as cathepsin B), accelerates trypsinogen activation, and thereby drives pancreatitis. At this stage, autophagy research was not yet decoupled from trypsin; rather, it took “the location at which trypsinogen activation occurs” as its entry point—autophagy or autophagy-like vacuoles were considered to potentially provide a platform for the abnormal co-localization of zymogen granules and lysosomal hydrolases, thereby promoting cathepsin B-mediated trypsinogen activation. Under this interpretation, the autophagy–lysosome system was positioned as the intra-acinar trypsin activation compartment—a functional module that promotes the pathogenic process.

#### 2.3.2 Conceptual shift: from trypsin activation compartment to homeostasis maintenance system

Mareninova et al. revised this picture by comparing the autophagic response in acute pancreatitis with the physiologic autophagy of starvation (Mareninova et al., 2009). In pancreatitis, autophagic vacuoles were more numerous and larger, degradation of long-lived proteins was suppressed rather than stimulated, and maturation of the lysosomal proteases cathepsin L and cathepsin B was impaired. Autophagosomes therefore accumulate because lysosomal degradation is deficient, not because autophagy is excessively induced—a distinction the elevated LC3-II and vacuole counts underlying the earlier “excessive autophagy” reading cannot capture, since impaired degradation and increased induction raise these markers alike. Because cathepsin B converts trypsinogen to trypsin while cathepsin L degrades both, defective cathepsin processing tips this balance toward intra-acinar trypsin accumulation; the reduced trypsinogen activation in autophagy-deficient mice thus reflects loss of a degradative system rather than removal of a pro-activation platform. A specific lesion in this pathway was identified by Fortunato et al. in a rat model of alcohol plus endotoxemia, in which depletion of LAMP-2—required for autophagosome–lysosome fusion—impaired autolysosome formation and accompanied a shift from apoptotic to necrotic acinar death; LAMP-2 depletion was also present in human alcoholic pancreatitis (Fortunato et al., 2009).

Taken together, these studies relocate the autophagy–lysosome system from a trypsin-activation compartment to a system for maintaining acinar homeostasis, shifting the explanatory frame from “autophagy promotes pathogenesis” to “deficient autophagic degradation drives pathogenesis.” This does not negate the Hashimoto phenotype—genetic loss of autophagy does reduce cerulein-induced trypsinogen activation and disease severity—but reassigns its mechanism, from removing a pathogenic platform to disabling a degradative system whose failure is itself injurious. The GEMM studies below sharpen the distinction.

#### 2.3.3 GEMM evidence for autophagy deficiency as an independent pathogenic mechanism

The reframing in Section 2.3.2 casts the autophagy–lysosome system as a homeostatic system of the acinar cell, but leaves one question untested: is deficient autophagic degradation sufficient to cause pancreatitis on its own, without an exogenous stimulus? Answering it requires conditional deletion of core autophagy genes in otherwise unchallenged pancreata.

Diakopoulos et al. addressed this with pancreas-specific *Atg5* deletion (*Ptf1a*-Cre^ex1^;*Atg5*^F/F^) (Diakopoulos et al., 2015). *Atg5*-deficient mice spontaneously developed an atrophic pathology resembling human chronic pancreatitis, inflammation, necrosis, acinar-to-ductal metaplasia, and tissue atrophy, with disease more pronounced in males than females. Mechanistically, ATG5 loss led to accumulation of reactive oxygen species, endoplasmic reticulum stress, mitochondrial damage, and p62 accumulation, with activation of p62/Nqo1/p53 signaling. The significance of this work is to move autophagy from a compartmental contributor to trypsinogen activation to a homeostatic requirement for maintaining acinar structure and function: even with trypsin displaced from the center, loss of autophagy is by itself sufficient to disrupt acinar homeostasis and produce a chronic-pancreatitis–like phenotype.

A design feature of this study deserves emphasis, because it governs how the Atg5 phenotype should be read: within the same work, the deletion was driven by two Cre systems acting at different times. *Ptf1a*-Cre initiates recombination in multipotent pancreatic progenitors during early embryonic development, so autophagy is lost before the acinar compartment has fully matured; the resulting chronic-pancreatitis–like phenotype may therefore reflect developmental defects superimposed on the loss of autophagy in mature acinar cells, rather than mature-cell autophagy failure alone. When Atg5 was instead deleted in fully differentiated adult acinar cells using the inducible Elastase-CreER, only scattered vacuolization appeared, without progression to chronic pancreatitis. Because it confines deletion to the adult compartment, Elastase-CreER is the more suitable tool for isolating the role of autophagy in the mature pancreas, and its milder phenotype is the more direct readout of adult-onset autophagy loss. Read together, the two drivers are complementary rather than contradictory: the adult-restricted phenotype confirms that autophagy is genuinely required for homeostasis in the mature acinar cell, whereas the fuller phenotype produced by *Ptf1a*-Cre should be interpreted with its developmental contribution in mind. In this model, therefore, the developmental timing of the deletion, not the loss of autophagy alone, shapes the severity of the phenotype, a caveat worth weighing when comparing autophagy GEMMs that rely on different Cre drivers.

A subsequent study by Antonucci et al. reached a parallel conclusion in a second autophagy gene (Antonucci et al., 2015) Pancreas-specific *Atg7* deletion (*Pdx1*-Cre;*Atg7^F/F^*) produced severe acinar degeneration, pancreatic inflammation, and extensive fibrosis. ATG7 loss caused ER stress, mitochondrial dysfunction, AMPK activation, and a marked reduction in protein-synthetic capacity accompanied by loss of rough ER, and triggered acinar-to-ductal metaplasia. The particular contribution of this work is to tie basal autophagy to the maintenance of the acinar cell’s high protein-synthetic capacity: by clearing misfolded proteins and damaged organelles, ongoing autophagy sustains the ER and protein-synthetic machinery on which the exocrine pancreas depends.

Together, these models indicate that basal autophagy is not merely disrupted in disease but continuously maintains acinar homeostasis under physiologic conditions, and that its loss alone, independent of exogenous injury, is sufficient to produce the cardinal phenotypes of pancreatitis. They also point to autophagy as a shared node rather than an isolated module: a single degradative failure simultaneously precipitates ER stress, protein-aggregate accumulation, mitochondrial dysfunction, and inflammation. This convergence supplies direct GEMM support for the positioning of the autophagy, lysosome system as a central element of acinar homeostasis, and anchors the network view developed in this review, that impairment of one homeostatic module propagates across several pathological processes.

#### 2.3.4 Component-level GEMM dissection of the autophagy–lysosome system

The autophagy–lysosome system is a multi-component complex network—including autophagosome formation (VMP1, ATG5, ATG7), autophagosome–lysosome fusion (LAMP-2), lysosomal hydrolase delivery (the M6P pathway), and lysosomal biogenesis (TFEB), among other levels. The work of Diakopoulos et al. and Antonucci et al. primarily targets the autophagosome formation stage, while component-specific GEMM work at other levels demonstrates that defects at different nodes of this system can each independently induce pancreatitis.

Defects at the autophagosome–lysosome fusion stage can be illustrated by the LAMP-2 whole-body knockout mice of Mareninova et al. (2015) The earliest lesion is acinar-cell vacuolization from impaired autophagic flux, progressing to trypsinogen activation, macrophage-predominant inflammation, and stellate-cell activation—a chronic-type profile, though overt fibrosis does not develop and the endocrine compartment is spared. Notably, the decline in LAMP-2 seen across pancreatitis models is itself traced to cathepsin B–mediated cleavage of the LAMP molecule, so that a protease central to trypsinogen activation also degrades a protein required for fusion—an early indication that the components of this pathway are not damaged in isolation but through shared mediators.

Lysosomal renewal converges on TFEB, whose degradation links acinar stress back to autophagic capacity. During cerulein pancreatitis, MTOR activation phosphorylates TFEB and drives its proteasomal degradation, lowering lysosome numbers until autophagy can no longer keep pace (Wang et al., 2019). Deleting *Tfeb* in acinar cells is not by itself sufficient for spontaneous disease but sensitizes the pancreas to cerulein, and only combined loss of *Tfeb* and *Tfe3* yields spontaneous pancreatitis—evidence that the two factors are partly redundant. That pharmacologic inhibition of either MTOR or the proteasome restores TFEB and limits damage places its loss upstream of the injury rather than alongside it.

Hydrolase delivery proves equally indispensable, and through an unexpected route. Ablating *Gnptab*, which tags newly made hydrolases for the lysosome via the M6P pathway, causes spontaneous pancreatitis whose proximate lesion is not proteolytic but lipid: free cholesterol accumulates in lysosomes and autolysosomes while the plasma membrane is depleted, autophagic flux stalls, and the cardinal features of disease follow (Mareninova et al., 2021). The liver is largely unaffected, marking this as an organ-specific reliance on M6P-dependent delivery. The remaining step, autophagosome closure, completes the pattern: acinar-specific loss of *Vmp1*, an ER membrane protein required to seal the nascent autophagosome, again produces a chronic-pancreatitis–like phenotype with inflammation, acinar-to-ductal metaplasia, and fibrosis, accompanied by ER stress, p62 accumulation, and NFE2L2/NRF2 activation—the last of which is pathogenic rather than protective here, since removing it relieves the disease (Wang S. et al., 2022).

What makes the series informative is less that each lesion causes pancreatitis than what the lesions share once they do. Across formation, fusion, delivery, and renewal, the same downstream signature recurs—stalled flux, p62 accumulation, ER stress, NRF2 activation, and a drift toward macrophage-driven inflammation—largely regardless of which component was removed. This convergence is what the network reading of acinar homeostasis would predict: the autophagy–lysosome system fails as a unit, because a defect at any one step is relayed through a common set of stress responses to the same pathological endpoint, rather than producing a lesion confined to the step that was lost.

### 2.4 Protein quality control: from acute UPR response to hereditary misfolding-induced pathology

The autophagy–lysosome system examined in Section 2.3 and the endoplasmic-reticulum (ER) protein-quality-control machinery considered here are not independent modules but two arms of a single, tightly coupled response to proteostatic stress. Persistent ER stress activates autophagy to remove damaged proteins and organelles, and functional autophagy in turn relieves the ER by clearing misfolded proteins and damaged ER membranes; conversely, defective autophagy exacerbates ER stress by allowing misfolded proteins to accumulate. Because a primary lesion in either pathway therefore propagates into the other, dysfunction of either one is sufficient to promote pancreatitis—which is also why the misfolding-driven models discussed below so consistently display combined ER-stress and autophagic signatures. Building on the autophagy GEMMs of Section 2.3, this section turns to the ER folding machinery itself and to the genetic evidence that failure of protein quality control constitutes an independent pathogenic module.

#### 2.4.1 Pancreatic protein synthesis burden and UPR as a research entry point

Among mammalian cells, pancreatic acinar cells carry one of the heaviest protein-synthetic loads, devoting the bulk of their biosynthetic output to secretory digestive enzymes. This places an unusually high demand on the endoplasmic reticulum (ER) and makes acinar cells more dependent on ER folding homeostasis than most cell types. When unfolded proteins accumulate in the ER—from elevated synthesis, reduced folding capacity, or production of folding-incompetent proteins—the cell mounts the unfolded protein response (UPR), a conserved program that restores folding homeostasis or, failing that, commits the cell to death.

In experimental pancreatitis the UPR is demonstrably engaged: in cerulein-induced and ethanol-associated injury, the kinetics of *Xbp1* splicing, ATF4 induction, and CHOP expression track the onset of acinar damage (Lugea et al., 2011; Sah et al., 2014). The UPR is transduced through three parallel sensor branches that act largely through transcriptional programs while also throttling new protein influx into the ER. PERK attenuates bulk translation through eIF2α phosphorylation, which simultaneously permits preferential translation of ATF4 and its targets CHOP and GADD34. IRE1α acts through two outputs: it catalyzes the non-canonical splicing of *Xbp1* mRNA to yield the transcription factor XBP1s—inducing ER-associated degradation (ERAD), lipid synthesis, and ER membrane expansion—and, through regulated IRE1-dependent decay (RIDD), degrades ER-bound mRNAs to lower folding load. ATF6, upon ER stress, traffics to the Golgi, where site-1 and site-2 proteases release a cytosolic fragment that enters the nucleus to upregulate ER chaperones such as GRP78/BiP together with ERAD components (Walter and Ron, 2011). Within a tolerable range the coordinated output of the three branches is adaptive; under severe or sustained stress the balance shifts toward apoptosis—a transition that for the PERK branch tracks the level of eIF2α phosphorylation, with CHOP among its pro-death effectors.

Whether the UPR engagement in pancreatitis is cause or consequence is harder to establish in these models, because acute injury collapses several homeostatic axes at once—the digestive-enzyme, inflammatory, and autophagic processes treated in Sections 2.1–2.3 are co-activated, and the specific contribution of ER stress cannot be cleanly resolved against that background. Isolating UPR dysregulation as an independent pathogenic module therefore requires perturbations that disrupt ER folding directly and genetically, rather than provoking ER stress as one consequence of acute injury—an approach for which human genetics provided the initial leads, developed in the sections that follow.

#### 2.4.2 Clues from human genetics: misfolding mutations as risk factors

The work of Witt et al. represents an important conceptual milestone in pancreatitis genetics (Witt et al., 2013). This study sequenced the *CPA1* gene (encoding carboxypeptidase A1) in patient cohorts with early-onset chronic pancreatitis and identified multiple heterozygous missense mutations (such as N256K, V251M), that were significantly associated with early-onset disease. Unlike *PRSS1*, *SPINK1*, and *CTRC*, *CPA1* does not act through trypsin: the authors found no effect on trypsinogen activation or on trypsin degradation by CTRC. Most pathogenic variants were instead loss-of-function, with apparent activity reduced by more than 80%; as apparent activity combines secretion, proteolytic stability, and catalysis, and the loss tracked markedly reduced secretion, the deficit reflects impaired secretion from a misfolded protein rather than loss of catalysis as such—though the assay does not fully separate the two. Consistent with misfolding, the most frequent variant (p.Asn256Lys) induced the ER stress markers *Xbp1* splicing, BiP, and calreticulin in AR42J pancreatic acinar cells. Given that proCPA1 is among the most abundant secreted pancreatic proteins, misfolding-induced ER stress is a plausible route from a heterozygous variant to chronic injury, independent of trypsin.

The principle extends to a second secretory enzyme. Fjeld et al. described CEL-HYB, a hybrid allele from non-allelic homologous recombination between *CEL* (carboxyl ester lipase, expressed mainly in acinar cells) and its pseudogene *CELP*, encoding a chimera with the CEL catalytic region but an aberrant *CELP*-derived C-terminus (Fjeld et al., 2015). The allele was enriched in familial and non-alcoholic chronic pancreatitis and, more modestly, in alcohol-related disease. In cellular models the protein showed reduced secretion and, on inhibition of synthesis, accumulated intracellularly far more than wild-type CEL; its lipolytic activity was lower, though the authors argued that altered activity does not drive disease. Notably, CEL-HYB elevated the autophagy marker LC3-II rather than ER stress markers, aligning it with the impaired autophagic flux of Section 2.3 rather than the UPR.

Two points raise these genes above a risk-gene list. Both act outside the protease–antiprotease system, and the genetics confirm their independence: pathogenic *CPA1* variants rarely co-occur with other susceptibility variants, and CEL-HYB carriers were essentially mutually exclusive with *PRSS1* mutation, unlike *CTRC*. Misfolding and defective secretion thus form a pathogenic route distinct from trypsin dysregulation. Yet the two diverge downstream—*CPA1* toward the UPR, CEL-HYB toward autophagy—so the shared lesion is best read not as one effector but as a sustained burden on the disposal of malformed proteins, a chronic, low-grade character matching the slow, recurrent, heritable profile of these forms.

#### 2.4.3 GEMM validation of misfolding mutations as independent causes of pancreatitis

Human genetic associations are correlative; demonstrating that misfolding itself causes disease requires reconstructing the variants in mice. Knock-in models across four secretory enzymes (*Cpa1*, *Cel*, *Ctrb1*, and *Pnlip*) now provide that evidence and reveal both a shared pathway and where the models diverge.

The CPA1 N256K knock-in mouse reported by Hegyi and Sahin-Tóth provided the foundational direct GEMM evidence (Hegyi and Sahin-Tóth, 2019). Homozygous mice develop spontaneous, progressive disease, with acinar atrophy, inflammation, fibrosis, and acinar-ductal metaplasia accompanied by modest BiP and marked CHOP induction (*Xbp1* splicing was seen only in cell culture). Two points are central. First, the ER stress is low-level: electron microscopy showed only occasional ER whorls without cisternal dilation, and apoptotic cells were scarce. Second, a *Cpa1*-null strain remained disease-free, establishing that the lesion is the misfolded protein rather than loss of function, a quality-control failure rather than altered trypsin activity.

To extend the CEL-HYB1 pathogenic mechanism from the human genetic correlative evidence of Fjeld et al. to GEMM-level causal validation, Mao et al. constructed humanized mice carrying the human CEL-HYB1 allele through CRISPR-Cas9 (Mao et al., 2022). Acini showed reduced secretion and insoluble aggregates with low-level ER stress. Autophagy was biphasic, activated when young and then failing in aged mice, where large autophagic vesicles accumulated with concurrent LC3-II and p62 elevation, a concrete *in vivo* link to the autophagy–lysosome axis of Section 2.3. The authors attributed the weaker phenotype, relative to *Cpa1*N256K, to CEL-HYB1 being a moderate risk factor rather than a strongly causative variant.

Regarding the misfolding pathogenic mechanism of the CTRB family, Bodas et al. constructed a mouse model carrying an exon 6 deletion (*Ctrb1^Δexon6^*) through CRISPR-Cas9 (Bodas et al., 2025). The human *CTRB2* exon 6 deletion it reproduces is a GWAS risk variant for pancreatic ductal adenocarcinoma, not an established chronic-pancreatitis allele, so the model probes proteotoxic ER stress at the cancer-risk end of the spectrum. At three months the pancreas was histologically normal, yet ultrastructure showed pronounced ER dilation with cytoplasmic and nuclear inclusions, and transcriptomics showed a downregulated acinar program with upregulated ER-stress and inflammatory pathways. Rather than causing overt pancreatitis, the mutation left acinar cells unable to recover from cerulein injury. Tauroursodeoxycholic acid or the anti-inflammatory sulindac partially relieved the phenotype, indicating that misfolding-driven ER stress is pharmacologically tractable. The *Pnlip* T221M model carries the principle to a pure lipase unrelated to trypsin (Zhu et al., 2023). Homozygotes develop the full hallmarks of chronic pancreatitis, heterozygotes more slowly. Mutant PNLIP accumulated as insoluble aggregates and produced overt ER stress (dilated ER, elevated *Hspa5*) and a maladaptive UPR, with *Ddit3*/CHOP upregulation, NF-κB activation, and acinar death. The NF-κB component is notable, because misfolding here engages the inflammatory module of Section 2.2 directly, not ER stress alone.

A parallel comparison of the *Cpa1* N256K and *Pnlip* T221M strains found that both reach chronic pancreatitis through largely overlapping pathways (ER stress, UPR activation, convergent death signaling), differing mainly in tempo and severity, with the *Pnlip* model the more aggressive (Wilhelm et al., 2026). Apoptosis was activated redundantly through intrinsic and extrinsic routes, and the authors concluded that this redundancy makes downstream death pathways poor drug targets, whereas reducing the upstream burden of misfolded enzyme is more tractable.

Across these models a consistent logic emerges, independent of trypsin activation or an exogenous trigger: a variant causes misfolding, misfolded enzyme accumulates in the acinar ER, and proteotoxic stress drives injury, inflammation, atrophy, and fibrosis. Notably, this module is not confined to non-trypsin enzymes: cationic trypsinogen itself misfolds pathogenically, through the human PRSS1 variants R116C and C139F and the orthologous mouse T7C140S knock-in (Section 2.1.3), so the same proteostatic failure seen for CPA1, CEL, CTRB, and PNLIP reaches the very enzyme at the center of the classical trypsin hypothesis (**Table 3**). The models also mark out real differences. Severity tracks how strongly causative the variant is, ER stress ranges from barely detectable (Cpa1 N256K) to overt (Pnlip T221M), and downstream output engages autophagy or NF-κB to varying degrees. How that injury is executed is less linear than the chain implies, which Section 2.4.4 takes up.

**Table 3:** Misfolding/proteotoxicity GEMMs.

Category	Mouse model	Main message	Ref.
Mild/moderate ER stress	CEL-HYB1	Moderate risk variant; age-dependent autophagy failure	Mao et al., 2022
Injury susceptibility	Ctrb1Δexon6	Increases injury susceptibility, not spontaneous disease	Bodas et al., 2025
Severe proteotoxicity (spontaneous chronic pancreatitis)	Cpa1 N256K	Misfolding alone, chronic pancreatitis; low ER stress	Hegyi and Sahin-Tóth, 2019
	Pnlip T221M	Misfolding alone, chronic pancreatitis; overt ER stress + NF-κB (most severe)	Zhu et al., 2023
	T7C140S (trypsinogen)	Even trypsinogen misfolds,chronic pancreatitis via ER stress, not activation	Sándor et al., 2025

#### 2.4.4 Complexity of downstream death execution: CHOP upregulation but not the sole driver

The misfolding models of Section 2.4.3 consistently showed upregulation of CHOP (DDIT3), the pro-apoptotic transcription factor downstream of the PERK–eIF2α–ATF4 arm, to which the ATF6 and IRE1 arms also contribute. Because CHOP is the classical effector that tips a sustained UPR from adaptation toward apoptosis, a reasonable inference was that it executes acinar death in the misfolding chain.

Németh et al. tested that inference directly (Németh et al., 2022). They crossed the *Cpa1* N256K strain with mice globally deleted for *Ddit3*/*Chop* and followed the course of disease. Despite clear *Ddit3* upregulation in *Cpa1* N256K pancreas, its deletion changed neither the onset nor the progression of pancreatitis: *Cpa1* N256K; *Ddit3*-KO mice developed disease with a time course and severity indistinguishable from the parent strain across atrophy, plasma amylase, histology, acinar-ductal metaplasia, macrophage infiltration, and fibrosis. *Ddit3*-KO mice alone showed no pancreatic pathology, excluding the possibility that injury from *Ddit3* loss itself masked a protective effect. The conclusion was that CHOP plays no significant role in misfolding-induced pancreatitis and is not a viable therapeutic target.

This refines, rather than overturns, the chain of Section 2.4.3. Misfolding still causes disease, but its downstream execution is not the linear UPR–CHOP apoptosis sequence the classical model implies. In *Cpa1* N256K mice the CHOP induction tracks ER stress without driving it, and the authors note two possibilities consistent with their data: the mild ER stress in this model may not be pathogenic, with misfolding acting through other routes; or aspects of ER stress not assayed here, such as calcium overload and ATP depletion, may carry the injury. They further suggest acinar death may not even be required to initiate disease, with stressed acini instead secreting factors that recruit macrophages and activate stellate cells.

The same caution applies to the other models. The *Pnlip* T221M pancreas shows CHOP upregulation alongside NF-κB activation (Zhu et al., 2023), but whether CHOP drives death there has not been tested by the reverse-genetic cross that would settle it, namely *Pnlip* T221M combined with *Ddit3*-KO. Given that diverse misfolding models share the CHOP signature, the most economical reading is that CHOP is a general marker of the ER-stress response, while the nodes that actually execute acinar death remain undefined and may differ among models. Németh et al. themselves point away from a single linear route, noting that the mild ER stress in *Cpa1* N256K mice may not be pathogenic, that aspects of ER stress not assayed there, such as calcium overload and ATP depletion, may carry the injury, and that acinar death may not even be required to initiate disease (Németh et al., 2022). The parallel comparison by Wilhelm et al. across the *Cpa1* N256K and *Pnlip* T221M models, which found apoptosis activated redundantly through intrinsic and extrinsic routes, is consistent with this view. More broadly, an elevated downstream marker in a misfolding model does not by itself identify the causal node. Distinguishing marker from driver requires gene deletion or pharmacological inhibition, as the *Ddit3* cross illustrates: the correlative observation that CHOP is upregulated in *Cpa1* N256K mice was tested directly and found not to be causal.

### 2.5 Calcium-mitochondria axis: from physiological calcium signaling to pathological metabolic collapse

#### 2.5.1 Acinar cell calcium signaling physiology as a research entry point

Acinar calcium signaling governs a range of cellular functions. Apical IP3 receptor (IP3R)-mediated Ca²^+^ oscillations drive exocytosis of zymogen granules, and mitochondrial Ca²^+^ uptake through the mitochondrial calcium uniporter (MCU) activates tricarboxylic-acid-cycle enzymes to sustain energy metabolism. The signal is spatially confined: a perigranular belt of mitochondria at the ER–apical boundary acts as a firewall that keeps physiological Ca²^+^ rises restricted to apical oscillations, while store depletion activates store-operated calcium entry (SOCE) through STIM1 and Orai1 to refill the ER.

Pancreatitis triggers, such as bile acids and fatty acid ethyl esters (FAEE, the non-oxidative metabolites of ethanol), can shift acinar Ca²^+^ signals from oscillatory to a sustained plateau, exceeding the processing capacity of the mitochondrial firewall and resulting in mitochondrial calcium overload. Whether this calcium disturbance is a passive consequence of injury or an independent trigger was addressed by intervention and genetic studies.

#### 2.5.2 Pathological calcium signaling as a pancreatitis causative factor: from mechanistic observations to human genetic clues

Calcium dysregulation contributes to pancreatitis through linked mechanisms. Frick et al. showed that elevated extracellular calcium did not by itself raise trypsinogen activation in rat acini, but markedly amplified the activation produced by secretagogue stimulation, an effect blocked by agents that lower cytosolic calcium (Frick et al., 1997). This places calcium upstream of the aberrant protease activation considered in Section 2.1.

Work from the Petersen and Criddle groups defined how the common clinical triggers act. In isolated mouse acinar cells, FAEE and fatty acids released ER calcium through IP3R and, after hydrolysis, impaired mitochondrial respiration and depleted ATP; the loss of ATP disabled the SERCA and PMCA calcium pumps, so cytosolic calcium remained elevated as a toxic plateau that depolarised mitochondria and drove necrosis (Criddle et al., 2006; Petersen, 2023). Supplying intracellular ATP restored pump function and prevented the plateau, identifying ATP-dependent pump failure rather than calcium release alone as the basis of the sustained signal. Petersen later integrated bile-acid and alcohol-related injury under the concept of toxic Ca²^+^ signals and extended it to stellate and immune cells.

Human genetic evidence came from whole-exome sequencing by Masamune et al. in early-onset chronic pancreatitis, which found functionally deficient variants of *TRPV6*, a highly calcium-selective channel expressed mainly in pancreatic ducts, significantly enriched in patients (Masamune et al., 2020). *TRPV6* joins *PRSS1*, *SPINK1*, *CTRC*, *CPA1*, and *CEL* as a susceptibility gene, but is the first that acts on calcium homeostasis itself rather than as a digestive enzyme or its inhibitor. The direction of effect is important: the variants are loss-of-function, and *TRPV6*^mut/mut^ mice developed more severe cerulein pancreatitis, indicating that functional TRPV6 is protective and that impaired calcium handling, not excess channel activity, confers risk. The association was replicated in a Chinese cohort, in Polish paediatric and German cohorts, and in French hereditary, familial, and idiopathic cases together with a four-population meta-analysis, with the variants most frequent in hereditary and familial disease (Zou et al., 2020; Oracz et al., 2021; Hamada et al., 2022). Across these studies the loss-of-function variants are frequently co-inherited with risk variants in *SPINK1*, *CTRC*, or *CFTR*, indicating that TRPV6 deficiency typically acts as part of a cumulative genetic burden rather than as a single-gene cause.

#### 2.5.3 GEMM validation of the calcium-mPTP axis: from CypD to Orai1 and TRPV6

Genetic and pharmacological studies have addressed both a downstream execution node of the calcium–mitochondria axis, the mitochondrial permeability transition pore (mPTP) regulated by cyclophilin D (CypD), and upstream plasma-membrane calcium channels, Orai1 and TRPV6.

The mPTP has been probed with complementary genetic and pharmacological tools. Mukherjee et al. tested cyclophilin D-knockout mice (*Ppif*^−/−^) across several acute pancreatitis models and found reduced pancreatic injury in each; the non-immunosuppressive CypD inhibitor alisporivir (DEB025) and the mPTP inhibitor TRO40303 gave similar protection in mouse and human acinar cells and *in vivo* (Mukherjee et al., 2016). In these models, toxin-induced calcium release through IP3 and ryanodine receptors opened the mPTP, collapsing ATP production and driving necrosis, which positions mPTP opening as a convergence point downstream of mitochondrial calcium overload and identifies CypD as a candidate drug target.

Upstream calcium entry has been addressed both pharmacologically and genetically. Wen et al. showed that selective Orai1 inhibitors (GSK-7975A and CM_128) blocked store-operated calcium entry and prevented necrosis in human and mouse pancreatic acinar cells, and reduced injury in three acute pancreatitis models (Wen et al., 2015). A selective Orai1/CRAC channel inhibitor of the same class, CM4620 (Auxora), has since been evaluated in a phase 2 trial in acute pancreatitis with systemic inflammatory response syndrome, with a favorable safety profile supporting further controlled study (Bruen et al., 2021). Genetic evidence comes from cell-type-specific deletion: pancreas-specific *Orai1* knockout reduced pancreatic tissue damage and immune-cell infiltration in experimental acute pancreatitis, whereas neutrophil-specific *Orai1* deletion instead reduced pancreatitis-associated lung injury, indicating that Orai1 drives pancreatic and systemic injury through different cell types (Niu et al., 2023). For TRPV6, Masamune et al. extended earlier work on global *Trpv6^mut/mut^* mice by generating pancreas-specific *Trpv6* conditional knockout mice (Pdx-1-Cre: *Trpv6^fl/fl^*), which developed more severe caerulein-induced acute and chronic pancreatitis than controls (Masamune et al., 2026). Caerulein upregulated TRPV6 in acinar cells, and deletion in ductal organoids had little effect, locating the protective function of TRPV6 in acinar rather than ductal cells. These findings establish TRPV6 as a protective channel and align with the human genetics, in which loss-of-function variants increase disease risk.

Taken together, these studies indicate that calcium handling acts on pancreatitis at more than one point. Excessive Orai1-mediated entry and loss of protective TRPV6 function both disturb acinar calcium homeostasis and can promote mitochondrial calcium overload, while CypD-dependent mPTP opening lies downstream, linking that overload to ATP depletion and necrosis. These channels do not act in the same direction: disease follows excess entry through Orai1 but loss of function of TRPV6, so the axis is better described as several calcium-handling lesions converging on mitochondrial failure than as a single linear chain. Section 2.5.4 considers how subsequent work refines this picture.

#### 2.5.4 Complexity of downstream execution mechanisms: refinements to the classical model from mcu and trpv6 studies

A strictly linear reading of Section 2.5.3 would predict that inhibiting any node in the calcium–mitochondria axis should lessen pancreatitis. Two GEMM studies show this does not hold uniformly. Chvanov et al. examined MCU-knockout mice (MCU^−/−^), expecting that removing the principal channel for mitochondrial calcium uptake would limit mPTP opening (Chvanov et al., 2020). Acinar cells from these mice showed markedly reduced mitochondrial calcium uptake and a strong fall in stimulus-metabolism coupling, yet across three acute pancreatitis models MCU knockout did not reduce histological or biochemical severity; protection *in vitro* was limited, reaching significance only for taurolithocholic acid sulfate-induced necrosis, and oedema in the fatty acid plus ethanol model was if anything higher. The authors attribute this to redundancy among toxin-triggered damaging mechanisms, a reported fall in mPTP calcium sensitivity in MCU-deficient mitochondria (allowing pore opening without a rise in matrix calcium), and a small residual non-MCU route of mitochondrial calcium entry. The result parallels Section 2.4.4, where deleting CHOP does not rescue CPA1 misfolding pathology: removing one prominent node need not block disease when parallel routes remain.

The second study concerns the direction of TRPV6’s effect. Using adeno-associated virus-mediated pancreatic *Trpv6* knockdown and the TRPV6 inhibitor SORC27, Han et al. found that reducing TRPV6 function protected against caerulein-induced acute pancreatitis, lessening mitochondrial depolarisation, trypsin activation and necrosis; TRPV6 expression and currents were raised in acinar cells of mice and patients, and TRPV6 contributed to store-operated calcium entry through interaction with STIM1 (Han et al., 2025). This is opposite to Masamune et al., where pancreas-specific deletion and global loss-of-function worsened disease (Masamune et al., 2026). Several differences may bear on this: full genetic deletion versus partial knockdown plus acute pharmacological block, so compensatory remodeling in the deletion models may not be reproduced; a possible dual role for TRPV6, supporting calcium homeostasis normally but feeding pathological entry once disease is established; and differences in setting, since Han et al. studied acute caerulein disease and note that the chronic-pancreatitis genetics of TRPV6 loss-of-function, and the larger role of ductal and stellate cells in chronic disease, lie outside their model. These are alternatives rather than a settled reconciliation; together they show the pancreatic role of TRPV6 is not captured by treating loss of function as uniformly protective or harmful.

Read together, these studies leave the core of the axis intact (sustained calcium elevation driving mitochondrial dysfunction and necrosis) while qualifying the status of individual nodes: whether MCU is required, and whether reducing TRPV6 helps or harms, depends on the model and on how the channel is perturbed. For therapy, the implication is not that upstream targets fail and downstream targets succeed. Acute pharmacological inhibition of upstream entry has been protective for both Orai1 and TRPV6, whereas constitutive whole-body deletion of an upstream node (MCU, or TRPV6 in the deletion models) can give complex or opposite phenotypes through compensation; CypD-directed mPTP inhibitors such as alisporivir (DEB025) and TRO40303 act at the downstream convergence point and were protective across several models (Mukherjee et al., 2016). The contrast with Section 2.4.4 is instructive: there the decisive intervention lies upstream, at the misfolded-protein burden, whereas here a downstream node, the mPTP, is the more robust target. Both modules argue for identifying the node that actually limits disease rather than the most conspicuous one.

### 2.6 From the trypsin-centric framework to acinar homeostasis network failure

Mechanistic study of pancreatitis first developed within the framework of digestive-enzyme safety. Across three protective tiers (control of trypsinogen activation through *PRSS1*/*T7* and the lysosomal activator cathepsin B; trypsin inhibition through *SPINK1*/*Spink3*; and trypsinogen/trypsin degradation through cathepsin L, CTRC and CTRB1), GEMMs established that pancreatitis can be driven by three corresponding classes of event: premature trypsinogen activation, failed trypsin inhibition, and failed degradation. This framework explains much of hereditary pancreatitis and early acinar injury, but the evidence assembled across the preceding modules indicates it cannot, on its own, account for inflammation amplification, switching of cell-death modality, organ failure, the acute-to-chronic transition, or the chronic pancreatitis driven by misfolding genes outside the protease–antiprotease system, such as *CPA1*, *CEL* and *PNLIP*.

These considerations support understanding pancreatitis as a failure of the acinar homeostatic network. Rather than a unidirectional cascade in which trypsin activation injures the acinar cell, ignites inflammation and drives organ failure, the network view holds that acinar homeostasis is maintained jointly by several semi-independent control systems (digestive-enzyme safety, NF-κB inflammatory transcription, autophagy–lysosomal degradation, ER protein quality control, and calcium–mitochondrial energy homeostasis), and that disease follows global destabilisation of this network rather than isolated failure of any one component. Early acute pancreatitis is concentrated in acinar cells and engages several axes at once (calcium overload and mitochondrial damage, aberrant trypsinogen activation, autophagy–lysosomal dysfunction, ER stress, NF-κB activation), then extends extracellularly through DAMP release, regulated cell death and inflammatory mediators that recruit immune cells and drive local and systemic inflammation. A defining feature is that the network can be entered at more than one relatively independent node: the decoupling of inflammatory transcription from trypsin activation (NF-κB activation does not require trypsin, and *T7*-deficient mice lose almost all trypsin activation yet retain inflammation), the misfolding genes outside the protease system, and the calcium-channel variants together show that biliary bile acids, alcohol and its FAEE metabolites, hereditary trypsin variants, misfolding variants and calcium-handling defects can each enter through a different node and initiate the same network. The claim is not to displace trypsin but to reposition it: trypsin activation remains a key node, reciprocally constrained with calcium and mitochondrial status, autophagic flux, ER quality control and NF-κB transcription, and the disease phenotype reflects the state of the whole network rather than any single node.

Such a model requires cross-module connections, and the autophagy–lysosome system is the clearest example, bridging three layers: in proteostasis, it clears misfolded proteins and unprocessed secretory cargo, interfacing with the ER unfolded protein response; in organelle maintenance, it clears damaged mitochondria by mitophagy, coupling to the calcium–mitochondrial axis; and in inflammatory regulation, impaired flux lets adapter proteins such as p62/SQSTM1 accumulate, influencing NF-κB, NRF2 and p53 signaling. The *Atg5*, *Vmp1* and *Ikkα* knockout models converge on aberrant p62 accumulation and, through it, downstream stress and transcriptional responses, implicating NRF2, p53 and ER stress to differing degrees across models (Li et al., 2013; Diakopoulos et al., 2015; Wang S. et al., 2022). The most direct demonstration of this coupling comes from Biczo et al. (2018): mitochondrial dysfunction acts upstream through CypD (*Ppif*)-dependent permeability-transition-pore opening, driven either by mitochondrial calcium overload or, in models such as L-arginine pancreatitis, by a calcium-independent route in which enhanced CypD–ATP synthase interaction suppresses ATP synthase activity; this blocks autophagic flux and induces ER stress and lipid-metabolism deregulation. Deletion of CypD abolishes these downstream phenotypes, and the autophagy enhancer trehalose, which does not act on mitochondria directly, markedly attenuates disease. The autophagy–lysosome system therefore cannot be assigned to one module: it connects protein quality control, organelle homeostasis, inflammatory signaling and cell-death decisions, which is why its destabilisation can both initiate pancreatitis and amplify injury across axes.

**Figure 1:**
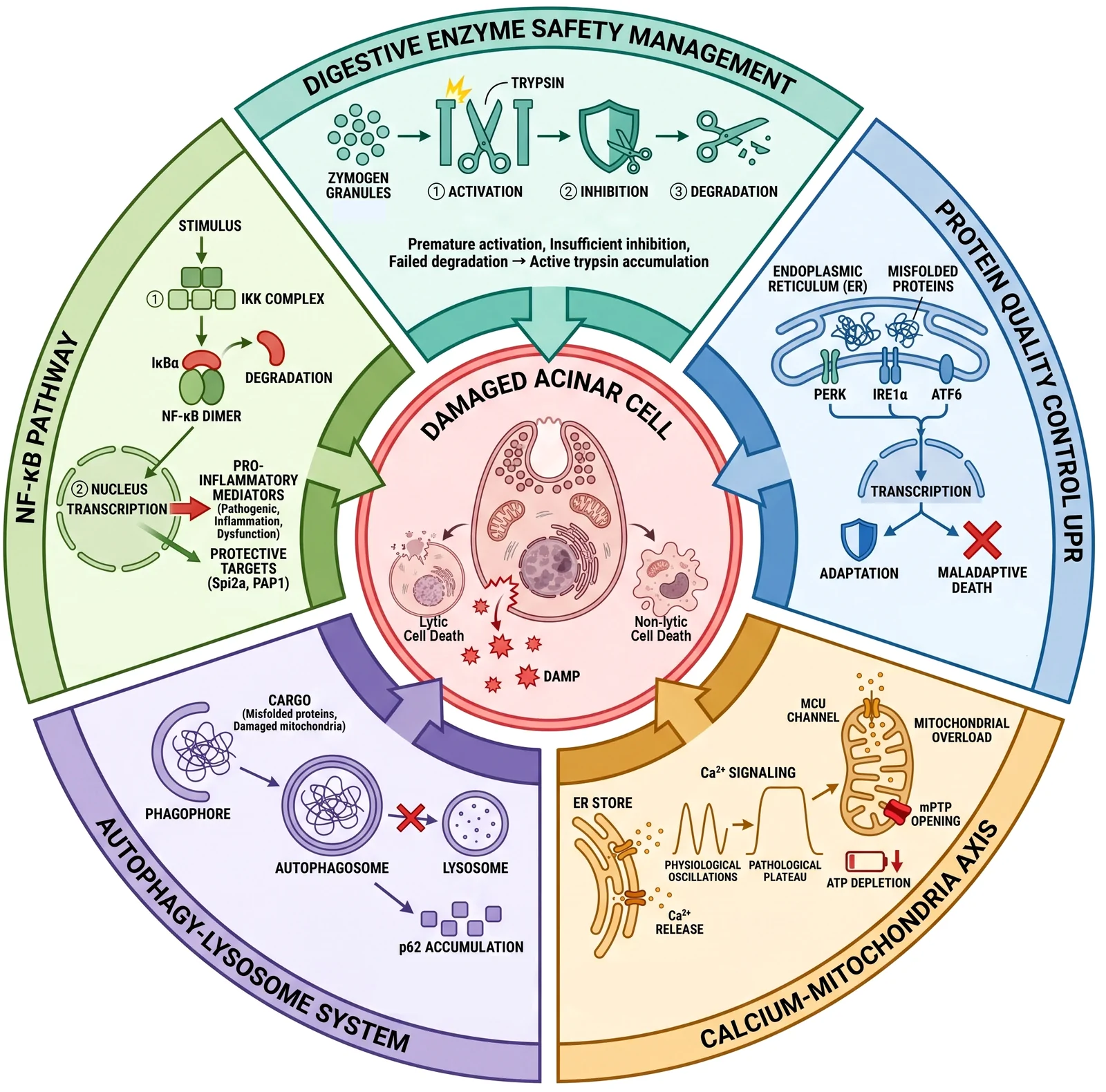
Pancreatitis as a failure of the acinar homeostatic network. Five control systems: digestive-enzyme safety, protein quality control (UPR), the calcium–mitochondria axis, the autophagy–lysosome system, and the NF-κB pathway jointly maintain acinar-cell homeostasis; destabilization of this network converges on the damaged acinar cell.

Across these modules, the evidence suggests pancreatitis is better understood as collapse of an interconnected acinar homeostatic network than as isolated failure of one enzyme or pathway. Different etiologies enter at different nodes and collapse along different trajectories, but these do not yield isolated outcomes: all drive the acinar cell through the same transition, from homeostasis into an injured state (**Figure 1**). What the network analysis explains is why and how the acinar cell moves toward injury; disease severity then rests on the fate decisions the cell makes once injured.

## 3 GEMM dissect the execution-pathway network of cell death: how the choice of death modality determines the severity of pancreatitis

### 3.1 From the injured state to execution trajectories: cell death modality as a determinant of disease severity

Entry into the injured state does not fix the fate of the acinar cell. The injured state at issue here is not a precise biochemical threshold or a single molecular event but a qualitative shift in the functional condition of the cell, recognizable operationally by several coexisting signatures: disruption of the secretory pathway (rearrangement of zymogen granules, aberrant intracellular activation, and leakage of zymogens into the cytosol), failure of energy metabolism (depletion of ATP reserves and mitochondrial dysfunction), and a pronounced rise in sensitivity to death-execution pathways, such that a death signal of given intensity elicits a stronger response than it would in the healthy cell. This last signature warrants emphasis: although heightened sensitivity lowers the threshold for death, it is only a priming condition and does not by itself dictate which trajectory the cell will follow, which is why a cell that has left the healthy state has not thereby had its outcome decided. Treating the injured state as a common point of departure has an analytical advantage: it accommodates heterogeneous trajectories of collapse, since whatever route brings the acinar cell to this state, the decisions that subsequently shape its execution trajectory follow broadly similar rules.

For decades, acinar cell death was framed as a binary of necrosis versus apoptosis. Because apoptosis is immunologically quiescent, it was regarded as the disease-limiting outcome, and therapeutic effort was directed at promoting it. This view has proven too simple. One influential line of evidence came from interspecies comparison: under identical cerulein stimulation, rats show robust caspase activation, prominent apoptosis, and mild self-limiting disease, whereas mice show suppressed caspase activity, predominantly necrotic death, and severe organ damage (Mareninova et al., 2006). This observation provided a direct indication that what shapes clinical outcome is how the acinar cell dies, not merely whether it dies (Sendler et al., 2016). The injured acinar cell thus faces not a binary of death versus survival but a set of competing execution trajectories: relatively silent clearance through apoptosis, in which membrane integrity is preserved; lytic death through necrosis, necroptosis, pyroptosis, or ferroptosis, in which the membrane ruptures and intracellular contents are released; or, where injury remains reversible, a return to homeostasis through regeneration and repair. It is this choice of trajectory, rather than the etiology that first drove the cell into the injured state, that largely sets disease severity, because the death modalities differ sharply in their consequences for DAMP release, inflammatory amplification, and tissue destruction.

As the understanding of regulated cell death (RCD) has deepened, this binary has given way to a more complex landscape. The Nomenclature Committee on Cell Death now recognizes more than a dozen RCD modalities (Galluzzi et al., 2018); of these, the ones with the strongest genetic and pharmacological support in experimental pancreatitis are apoptosis, necroptosis, pyroptosis, and ferroptosis, while PANoptosis, which combines features of pyroptosis, apoptosis, and necroptosis, has been proposed as an additional execution form in severe disease. These pathways do not operate in isolation. They share upstream triggers (calcium signaling and mitochondrial dysfunction, for instance, can engage several at once), cross-talk at the molecular level (caspase-8, for example, governs the choice between apoptosis and necroptosis), and compensate for one another at the phenotypic level (blockade of one pathway frequently redirects the cell toward another). For this reason, this section treats the death decision of the injured acinar cell not as a menu of independent options but as an interconnected network of execution pathways, in which severity is set not by any single pathway but by the configuration of the network as a whole.

### 3.2 Apoptosis and necroptosis: the classical death-mode switch

Apoptosis and necroptosis are mechanistically distinct but linked through a shared node, and genetically engineered mouse models have been the principal means of resolving how that node shapes pancreatitis. Apoptosis depends on a caspase cascade initiated through the mitochondrial or death-receptor pathway and converging on caspases-3 and -7, with the plasma membrane preserved. Necroptosis is caspase-independent, executed by the RIPK1–RIPK3 axis through MLKL-mediated membrane permeabilization and DAMP release. The connecting node is caspase-8: when active, it drives apoptosis while cleaving and inactivating RIPK1 and RIPK3 to restrain necroptosis; when its activity is lost, RIPK1 and RIPK3 assemble the necrosome. This reciprocity renders the two modes largely mutually exclusive within a cell and makes caspase-8 a paradigmatic shared node in the execution network.

That modality, not the mere occurrence of death, governs severity is illustrated by the rat–mouse comparison above: under identical cerulein stimulation, the caspase-competent rat develops mild, apoptosis-predominant disease and the caspase-restrained mouse severe, necrosis-predominant disease (Mareninova et al., 2006). Whether this regulated necrosis corresponds molecularly to necroptosis was then probed by gene knockout.

The knockout studies have not converged, and a feature of construct design is central to the discrepancies: the *Ripk3*, *Mlkl*, and *Saa3* deficiencies below were mostly whole-body (germline) knockouts. Some support a severity-driving role: in the models of Louhimo et al., necroptosis predominated with little apoptosis, and *Ripk3* deletion or necrostatin reduced severity in both cerulein- and bile-acid (TLCS)-induced pancreatitis (Louhimo et al., 2016); and serum amyloid A3 promotes RIPK3-dependent necroptosis upstream, so *Saa3* deficiency attenuated pancreatic and pancreatitis-associated lung injury (Yang et al., 2021). A second study, however, found no protection from *Ripk3* deficiency in the cerulein model (Newton et al., 2016). Others point the opposite way: deficiency of *Ripk3* or *Mlkl* instead aggravated disease, with *Mlkl* deficiency showing lower induction of the antiapoptotic genes *Bclxl* and *Cflar* and more apoptotic cells (Boonchan et al., 2021); and *Ripk3* deletion on a pancreas-specific autophagy-deficient (*Atg7*-knockout) background aggravated apoptotic acinar cell death and accelerated animal death (Zhou et al., 2017).

These conflicts can be read on two levels, both intrinsic to the models. The first is molecular: RIPK3 and RIPK1 have necroptosis-independent functions, including roles in caspase-8 activation and NF-κB signaling (Newton et al., 2016; Zhou et al., 2017). Deleting *Ripk3* therefore does not selectively remove necroptosis but also perturbs apoptosis and inflammation, which is why *Ripk3* and *Mlkl* deficiencies need not be equivalent (Newton et al., 2016); consistently, *Ripk3* deletion is protective in cerulein pancreatitis whereas pharmacological RIP1 inhibition aggravates it, so even within the necroptotic machinery RIPK1 and RIPK3 act inconsistently (Linkermann et al., 2012). The second is model design: because RIPK3 and MLKL operate in both acinar and immune cells, a whole-body knockout removes the function from both compartments, and the phenotype is their net—liable to shift or reverse with model, strain, and severity when the two oppose each other. Resolving this requires pancreas-specific conditional knockouts (e.g., via Cre-lox), which the current whole-body literature lacks.

The inconsistencies thus support this section’s thesis rather than undermining it: the execution pathways are interconnected and redundant, so removing one node often reroutes the death flux rather than abolishing a function. Necroptosis is accordingly neither simply injury-driving nor protective; its net effect depends on its coupling to apoptotic and inflammatory signaling in a given model, background, and compartment.

This balance can also be tested therapeutically. In mouse models of acute pancreatitis, the BCL2 inhibitor venetoclax shifted death from necrosis toward apoptosis, reducing severity and improving acinar cell viability and ATP (Litewka et al., 2025)—a proof of concept that shifting modality improves outcome, though the benefit was stage-dependent: venetoclax did not improve chronic pancreatitis, and the non-selective inhibitor navitoclax aggravated fibrosis. The ATP recovery is mechanistically informative: it was attributed to normalization of pathological Ca²^+^ signaling, which lowers the demand on ATP-dependent Ca²^+^ pumps, linking the death decision to the calcium–mitochondria axis of sectionr 3. Because apoptosis is itself energy-dependent, the ATP depletion driven by that Ca²^+^ overload biases the cell away from apoptosis and toward necrosis—so the mitochondrial and energetic state actively conditions the downstream choice. In sum, apoptosis and necroptosis form a caspase-8–gated switch that varies with the cell’s energetic and signaling state and can be shifted pharmacologically; it is both a determinant of severity and the earliest-established instance of the execution network.

### 3.3 Pyroptosis: inflammasome- and GSDMD-mediated pro-inflammatory death of acinar cells

Pyroptosis is a pro-inflammatory mode of regulated cell death that depends on caspase-1 and is executed by gasdermin D (GSDMD). Active caspase-1 processes pro-IL-1β and pro-IL-18 and cleaves GSDMD, releasing an N-terminal fragment that forms plasma-membrane pores, causing osmotic lysis and release of mature IL-1β and IL-18 (Shi et al., 2015). Unlike the immunologically silent clearance achieved by apoptosis, pyroptosis couples cell death to mediator release in a single event, making it at once lytic and strongly immunogenic. Acinar cells express the inflammasome components and can undergo cell-autonomous pyroptosis in injured states such as trypsinogen activation and calcium overload, while DAMPs released on lysis activate the inflammasome in neighboring cells, forming a self-amplifying circuit. Pyroptosis therefore sits at the junction where local acinar injury proceeds to systemic inflammation, and the evidence that acinar-intrinsic pyroptosis drives disease rests on compartment-specific genetic models.

Genetic dissection began with whole-body deletion of core components. Hoque et al. established the necessity of the pathway: in the cerulein model, deletion of NLRP3, ASC, or caspase-1 reduced pancreatic oedema, inflammation, and pro-IL-1β, as did deletion of the upstream DAMP sensors TLR9 and P2X7, and a TLR9 antagonist extended this protection pharmacologically to a biliary model (Hoque et al., 2011). Whole-body deletion, however, cannot identify the cell in which a gene acts; since NLRP3 is expressed in both acinar and immune cells, such models show that the inflammasome is required but not where the driving pyroptosis originates. The expression data of Hoque et al. in fact localized TLR9 mainly to resident macrophages, casting the acinar cell as the DAMP source. Resolving this attribution was the task of the conditional-knockout studies that followed.

Gao et al. addressed it with cell-specific knockout. Deletion of *Nlrp3* or *Gsdmd* was restricted to acinar cells with Pdx1-Cre, and deletion of *Gsdmd* separately to the myeloid compartment with Lyz2-Cre, allowing a direct comparison at the level of GSDMD. Pancreas-specific deletion attenuated acinar pyroptosis, pancreatic necrosis, and systemic inflammation, whereas myeloid deletion of *Gsdmd* did not, indicating that the principal driver is acinar-intrinsic pyroptosis rather than pyroptosis in immune cells (Gao et al., 2021). Two further observations followed: deletion of *Gsdmd* alone preserved acinar cells, identifying pore formation as an obligatory execution step; and combining a RIPK3 inhibitor with *Gsdmd* deficiency gave greater protection than blocking pyroptosis alone, pointing to crosstalk with necroptosis at the level of execution. Consistent with this assignment, Lu et al. used the same Pancreas-specific knockout mice to show that the protection afforded by HDL and its apolipoprotein apoA-I depends on acinar NLRP3 and GSDMD, since deleting these components abolished it (Lu et al., 2023).

The intensity of pyroptosis is set not by the inflammasome alone but by upstream signals corresponding to the acinar homeostatic modules of section 3, defined across different systems and species. Cathepsin B released on lysosomal destabilization acts upstream of the inflammasome, since in mice its inhibition reduces NLRP3 and caspase-1 whereas caspase-1 inhibition does not affect cathepsin B, linking pyroptosis to the autophagy-lysosome pathway of Section 2.3 (Wang et al., 2021). Mitochondrial quality control forms a second checkpoint: mice lacking the mitophagy genes *Pink1* or *Park2* show enhanced inflammasome activation and aggravated disease from defective clearance of damaged mitochondria, reversed by the NLRP3 inhibitor MCC950 and echoing the calcium and mitochondria axis of Section 2.5 (Zhang et al., 2021). Endoplasmic reticulum stress promotes caspase-1-dependent acinar pyroptosis through the PERK branch, connecting with the protein-load theme of Section 2.4, although this link was established in a rat model with lentiviral knockdown rather than in knockout mice (Yan et al., 2023). The execution of pyroptosis is thus embedded in the organellar and metabolic state of the acinar cell.

The immunogenicity of pyroptosis extends its influence to systemic immunity. Sendler et al. found that the systemic inflammatory response syndrome (SIRS) and the compensatory anti-inflammatory response syndrome (CARS) do not arise sequentially but develop in parallel early in disease, both depending on NLRP3; inflammasome-derived IL-18 induces a Th2 response in the absence of IL-12 and bridges local injury to systemic immune dysregulation (Sendler et al., 2020). As in the work of Hoque et al., the inflammasome here localized to infiltrating macrophages rather than acinar cells, indicating that the systemic arm is executed largely in the immune compartment even when the initiating injury is acinar.

Finally, therapeutic testing examined whether the pathway can be interrupted pharmacologically. Zhao et al. showed that the approved drug disulfiram, by inhibiting GSDMD pore formation, reduced pancreatic cell death and multi-organ injury in a mouse model of severe pancreatitis, identifying the execution step as a tractable node (Zhao et al., 2025). As with the apoptosis and necroptosis balance in Section 3.2, intervening on an execution pathway both confirms the causal role of the corresponding death mode and points to the therapeutic potential of redirecting it. Pyroptosis thus deepens the section’s thesis on two levels: methodologically, acinar versus myeloid conditional knockouts resolved the driving pyroptosis to the acinar compartment, a resolution beyond the reach of whole-body deletion; mechanistically, its immunogenicity and its execution-level overlap with necroptosis mark it as a node within an interconnected death network rather than an isolated switch.

### 3.4 Ferroptosis: iron-dependent lipid peroxidation and the intrinsic vulnerability of acinar cells

Ferroptosis is an iron-dependent, caspase-independent execution pathway, and a series of genetically engineered mouse models targeting the acinar cell have advanced it from correlative description toward causal validation. Its executing event is the lethal peroxidation of membrane phospholipids (Dixon et al., 2012). The defensive core is the system xc−–glutathione (GSH)–GPX4 axis: xCT (SLC7A11) imports cystine for GSH synthesis, and GPX4 uses GSH to reduce phospholipid hydroperoxides to alcohols. When GPX4 is inactivated through transcriptional suppression, protein degradation, or GSH depletion, hydroperoxides accumulate, ferrous iron propagates lipid radicals through the Fenton reaction, and ACSL4 esterifies polyunsaturated fatty acids into membranes as peroxidation-prone substrates (Doll et al., 2017). The pathway is mechanistically distinct from the modes of the preceding section, being blocked neither by caspase inhibitors nor by *Ripk3* deletion. Acinar cells are intrinsically vulnerable to it: as one of the most biosynthetically active cell types, they sustain high oxidative phosphorylation to support enzyme secretion, generating reactive oxygen species as a constant by-product, a state in which perturbations of antioxidant defense that are tolerable elsewhere may become catastrophic. Whether this holds requires testing *in vivo*.

Two knockout models address the protective axis from its import and detoxification ends. At the import end, Pan et al. combined gene silencing, pharmacological inhibition, and whole-body *Slc7a11* knockout: silencing or inhibiting xCT in acinar cells lowered GSH, increased lipid ROS, and promoted ferroptosis, effects rescued by N-acetylcysteine or ferrostatin-1, while whole-body *Slc7a11* deletion increased acinar lipid peroxidation in the cerulein model (Pan et al., 2023). At the detoxification end, a study using pancreas-specific *Gpx4* knockout provided clearer cellular attribution: *Gpx4*-deficient mice developed more severe pancreatitis after cerulein or ethanol challenge, indicating that GPX4-mediated clearance of phospholipid hydroperoxides limits acinar injury (Liu et al., 2022). The difference in construct is itself informative: compared with the whole-body knockouts on which the necroptosis studies of the preceding section largely relied, pancreas-specific knockout localizes the injury-promoting effect of ferroptosis more clearly to the acinar compartment, partly addressing the compartment-attribution problem of Section 3.2. Together the two models give bidirectional genetic support for the SLC7A11–GSH–GPX4 axis in acinar ferroptosis.

The same *Gpx4* model revealed a direct interface between ferroptosis and a process specific to pancreatitis. Supraphysiological trypsin alone did not induce appreciable acinar death but markedly sensitized cells to ferroptosis triggered by cerulein, L-arginine, ethanol, erastin, or RSL3, through PSMD4-dependent lipid peroxidation that promoted proteasomal degradation of GPX4 (Liu et al., 2022). This connects the digestive-enzyme homeostasis of section 2 with the death-mode decision of this section: trypsin, which defines acinar identity, can itself weaken antioxidant defense under stress and bias the cell toward ferroptosis, giving a molecular basis for the heightened sensitivity of the injured state. By screening more than a thousand approved drugs, the study further identified the antipsychotic olanzapine as an antioxidant that inhibits acinar ferroptosis and attenuates experimental pancreatitis in *Gpx4*-deficient mice, a pharmacological reversal indicating that the ferroptosis seen in these models drives injury rather than merely accompanying it.

Susceptibility to ferroptosis is further regulated by upstream programs, also shown by conditional knockout. Pancreas-specific deletion of *Arntl*, encoding the circadian factor BMAL1, made mice develop L-arginine-induced pancreatitis more rapidly and with higher mortality, with aggravated tissue injury, neutrophil infiltration, and HMGB1 release; BMAL1 loss lowered the expression of antioxidant and repair genes including *Slc7a11* and *Gpx4*, and liproxstatin-1 reversed the aggravated injury, confirming ferroptosis as the intermediary (Liu et al., 2020). Acinar resistance to ferroptosis is thus under circadian control, a further upstream variable biasing the downstream choice of death mode in keeping with the logic of Section 3.2.

For the central thesis of this section, ferroptosis shows that the execution trajectory an acinar cell follows depends not only on the death signal but on its membrane-lipid and iron homeostasis and its capacity to clear lipid peroxides. Weakening cystine import through system xc− or deleting pancreatic *Gpx4* both render acinar cells more prone to ferroptosis and aggravate disease, and the protection afforded by ferroptosis inhibitors across models supports its causal status pharmacologically. Ferroptosis therefore links the upstream metabolic network of section 2 with the downstream mode of death, constituting a further node of the execution-pathway network, one rooted in the membrane-lipid and metabolic characteristics of the acinar cell.

### 3.5 PANoptosis—coordinated multi-modal cell death in pancreatitis

PANoptosis is a more recently proposed concept of inflammatory regulated cell death. In contrast to the view that treats pyroptosis, apoptosis, and necroptosis as independent pathways, it emphasizes that these three modes can be mobilized in an integrated manner through a macromolecular platform termed the PANoptosome, manifesting as a multi-modal death combining features of all three (Samir et al., 2020). The defining criterion is not whether the three occur simultaneously, but that the death possesses molecular and phenotypic features of all three that can be difficult to account for by any single pathway. This platform is typically nucleated by innate immune sensors, among which ZBP1 has been well characterized. ZBP1 recognizes Z-form nucleic acids through its Zα domains; beyond viral nucleic acids, Z-form nucleic acids arising from endogenous retroelements can also activate ZBP1 and trigger RIPK3-dependent death and inflammation under conditions of impaired RIPK1 or caspase-8 function (Jiao et al., 2020). By analogy, this raises the possibility that endogenous nucleic acids released by stressed or mitochondrially damaged acinar cells might act as a trigger for ZBP1 in the pancreas, although this remains an inference awaiting direct verification. Once activated, ZBP1 can recruit ASC, RIPK1, RIPK3, FADD, caspase-1, and caspase-8 into a signaling scaffold; AIM2, pyrin, and ZBP1 have been reported to form a multi-sensor PANoptosome (Lee et al., 2021), and NLRC5 has been reported to nucleate a distinct PANoptosome (Sundaram et al., 2024), indicating that the platform may represent an expanding family of sensors. Downstream execution is carried out by GSDMD, GSDME, and MLKL, with their relative contributions varying by cellular context. PANoptosis thus places the execution pathways of the preceding sections within a framework in which they are interwoven and can be mobilized together by a shared upstream event.

This framework may help interpret a recurring observation from the preceding sections: blockade of any single execution pathway tends to confer incomplete protection or to redirect death flux toward other pathways, as in the discordant necroptosis-knockout results of Section 3.2 and the additive protection from *Gsdmd* deficiency combined with RIPK3 inhibition in Section 3.3. If multiple execution modes are driven cooperatively by a shared platform, this admits a more unified explanation: after inhibition of one execution protein, the platform may complete death through the remaining executors. On this logic, targeting upstream nodes of the platform such as the sensors or the RIPKs has been proposed as a therapeutic strategy (Pandeya and Kanneganti, 2024). Caspase-8 again occupies a central position in this coordination, having been described as a molecular switch among apoptosis, necroptosis, and pyroptosis (Fritsch et al., 2019), which echoes its treatment as a shared node in Section 3.2. In this sense PANoptosis can be read as a fuller illustration of this section’s network thesis: the execution pathways are interconnected and, under certain conditions, may be mobilized together by a shared upstream signal.

Among studies linking PANoptosis to pancreatitis, the work of Li et al. is representative. That study used bioinformatic analysis of the pancreatic transcriptome in acute pancreatitis, found enrichment of PANoptosis-related pathways, and identified *Zbp1* among the hub genes; in cerulein-induced murine pancreatitis it then observed concurrent upregulation of multiple death markers, including activated caspase-1, cleaved caspase-3 and total caspase-8, phosphorylated RIPK3 and MLKL, and cleaved GSDMD, while knockdown of *Zbp1* downregulated these markers together and attenuated pancreatic inflammation, oedema, and acinar death (Li et al., 2025). Consistent with this, the natural product eriodictyol has been reported to attenuate inflammation and necrosis in experimental pancreatitis through ZBP1-dependent signaling, offering pharmacological corroboration (Tian et al., 2025). The strength of this evidence nonetheless differs from that of the preceding sections, on several counts. The interventions on ZBP1 were largely pharmacological inhibition or siRNA knockdown rather than knockout in mice, and so do not yet constitute definitive genetic evidence for a role of the PANoptosome in acinar cells. The relevant studies have relied largely on the cerulein model and the 266–6 cell line, whose equivalence to primary acinar cells is uncertain. And co-upregulation of multiple death markers at the tissue level does not by itself distinguish genuine concurrence of the three execution modes within a single cell from parallel activation of separate pathways in distinct cell subpopulations.

The relevance of PANoptosis in pancreatitis may extend beyond acinar cells. In the vascular endothelium, Ge et al. found that CIRP, a DAMP elevated in acute pancreatitis, induces PANoptosis markers in endothelial cells, with the natural product costunolide attenuating endothelial PANoptosis and pancreatitis-associated acute lung injury through ALDH2 (Ge et al., 2025); Liu et al. reported that targeting the TMAO–HMGB1 pathway suppresses endothelial PANoptosis and alleviates the lung injury of severe pancreatitis (Liu et al., 2026). In the immune compartment, Wu et al. used a macrophage membrane-coated nanocarrier to inhibit ZBP1-associated macrophage PANoptosis and thereby attenuate severe pancreatitis (Wu et al., 2026). These studies connect PANoptosis with the systemic complications of pancreatitis, particularly acute lung injury, suggesting it may not be confined to one cell type. This evidence again rests largely on pharmacological tools such as natural products and nanomedicines rather than genetic models; taken together with the studies above, it indicates that PANoptosis in pancreatitis is at present supported more by pharmacological inhibition and marker co-occurrence than by direct genetic manipulation.

With respect to this section’s thesis, PANoptosis can be regarded as a relatively full expression of the execution-pathway network: the modes of death are interconnected and able to compensate for one another to some degree, and under certain conditions may be mobilized together by a shared upstream signal, which offers a unified reading of the incomplete protection from single-pathway inhibition seen earlier. The causal role of the ZBP1-PANoptosome has received stronger genetic support in other diseases; in β-coronavirus infection, for instance, deletion of *Zbp1* or *Ripk3* reduces the activation of PANoptosis and the associated tissue injury under interferon treatment (Karki et al., 2022). By comparison, and to the best of current knowledge, the pancreatitis field still lacks knockout studies targeting *Zbp1* or other PANoptosome components, whether whole-body or pancreas-specific, with existing conclusions resting largely on bioinformatics and on pharmacological inhibition or knockdown. Consequently, set against the genetic resolution achieved for pyroptosis through pancreas-specific conditional knockouts and for ferroptosis through pancreas-specific *Gpx4* knockout, the evidence for PANoptosis in pancreatitis remains relatively preliminary; separating genuine multi-modal death at the single-cell level from pathway co-occurrence at the population level will likely require a combination of single-cell-resolution and genetic approaches. PANoptosis may thus be seen as one current frontier of this section’s model-driven account: its core machinery has been genetically validated in other diseases, whereas genetic evidence of comparable rigour in pancreatitis remains to be obtained.

### 3.6 Synthesis: execution pathways as an interconnected, switchable network

**Figure 2:**
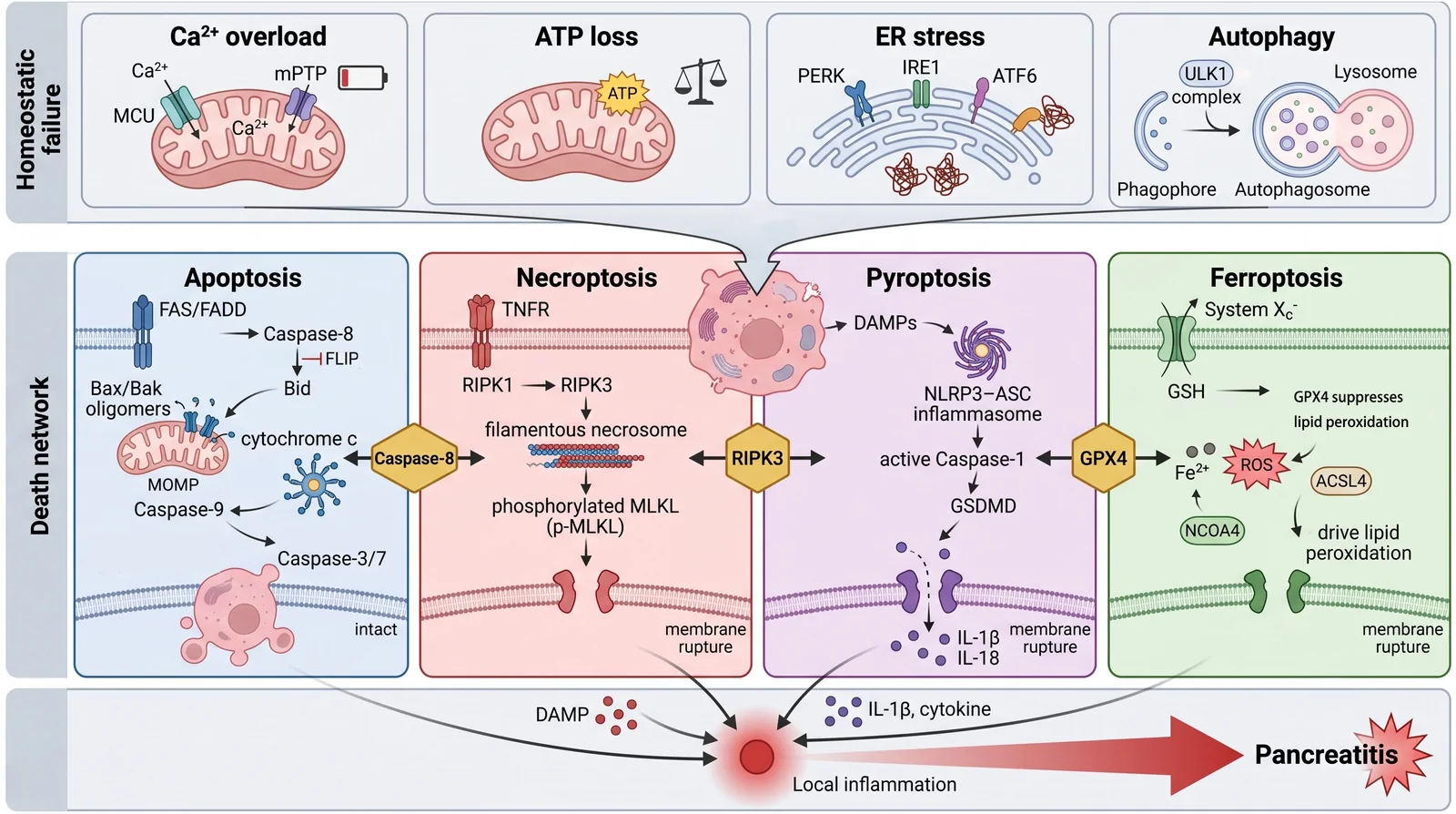
Upstream homeostatic failure drives an interconnected, switchable acinar cell-death network that converges on pancreatitis. Calcium overload, ATP depletion, ER stress (UPR) and autophagy–lysosome dysfunction converge on a damaged acinar cell and engage four regulated cell-death routes: apoptosis (non-lytic, membrane intact) and the lytic routes necroptosis, pyroptosis and ferroptosis (membrane rupture). These routes are interconnected through the shared nodes caspase-8, RIPK3 and GPX4, so that blocking one route reroutes the death flux to another; membrane rupture with release of DAMPs and cytokines then drives local inflammation and, ultimately, pancreatitis.

The preceding sections used genetically engineered mouse models to dissect, in turn, apoptosis and necroptosis, pyroptosis, ferroptosis, and their attempted unification under PANoptosis. A recurring pattern emerges: these execution pathways rarely behave as independent switches. Genetic or pharmacological perturbation of any single pathway tends to fall short of clean protection, with results that are discordant (as in the necroptosis knockouts), partially effective, or compensated for by other pathways. This section therefore advances the section’s integrative thesis: the death-execution pathways of the acinar cell are less a set of parallel programs than an interconnected network that can, under certain conditions, switch between its routes (**Figure 2**).

The interconnection is reflected first in the sharing of molecular nodes (**Figure 2**). Caspase-8 governs both apoptosis and necroptosis and serves as a node between them (Section 3.2); RIPK3 inhibition on a *Gsdmd*-deficient background confers additive protection (Gao et al., 2021), indicating that pyroptotic and necroptotic execution proceed through partly independent routes that converge on acinar necrosis (Section 3.3). A comparable node links ferroptosis and pyroptosis: in a sepsis model, myeloid-specific *Gpx4* deficiency enhances caspase-11 activation and GSDMD cleavage through a lipid-peroxidation-dependent route, making lipid peroxidation, the process central to ferroptosis, an upstream driver of pyroptotic execution (Kang et al., 2018); and in pancreatitis, wedelolactone attenuates both pyroptosis- and ferroptosis-related injury by upregulating GPX4, with the pyroptosis inhibitor disulfiram and the ferroptosis inhibitor ferrostatin-1 each conferring protection, suggesting that GPX4 acts as a shared regulatory node between the two (Fan et al., 2021). The interconnection extends to upstream homeostatic processes: ferritinophagy, the NCOA4-mediated autophagic degradation of ferritin, releases stored iron that fuels lipid peroxidation, and in severe pancreatitis silencing of *Ncoa4* ameliorates disease whereas its overexpression aggravates it (Lu et al., 2025); relatedly, extracellular SQSTM1 increases acinar susceptibility to ferroptosis by upregulating ACSL4 in an AGER-dependent manner (Yang et al., 2023). The autophagy-lysosome module (Section 2.3) is thus tied to the execution network through ferroptosis.

A second property is switchability: when one route is blocked, the death flux is often rerouted to completion rather than abolished. Broad caspase inhibition shifts acinar cells from apoptosis toward necrosis and aggravates disease (Mareninova et al., 2006); knockout of *Ripk3* or *Mlkl* yields discordant outcomes across studies, owing in part to such redistribution and to the non-necroptotic functions of RIPK1 and RIPK3; and *Gsdmd* deficiency alone often requires concurrent RIPK3 inhibition for further benefit (Gao et al., 2021). What determines outcome, then, is not whether a single pathway is activated, but how the network configures its death flux. That configuration is set upstream, by the homeostatic state of the acinar cell (section 2): energy and mitochondrial state bias the apoptosis-versus-necrosis choice, since ATP depletion can leave a cell unable to execute apoptosis and divert it toward necrosis; phospholipid composition and iron homeostasis set ferroptosis susceptibility; and lysosomal, mitophagic, and endoplasmic reticulum stress modulate pyroptosis. The central thread of sections 2 and 3 is therefore that severity reflects not any single death pathway but a network configuration rooted in the upstream homeostatic network.

This perspective carries two implications. For therapy, it helps explain the limited benefit of single-pathway interventions and suggests that targeting shared nodes such as caspase-8, RIPK3, or GPX4, or the upstream homeostatic determinants, might hold more promise; it should be read with caution, however, that much of the present crosstalk evidence is pharmacological, and the full network is still being assembled. The network is also likely larger than currently depicted: the landscape of regulated cell death has expanded to include cuproptosis (Tsvetkov et al., 2022), disulfidptosis (Liu et al., 2023), and lysosome-dependent cell death, whose roles in pancreatitis remain largely undefined, yet the metabolic profile of acinar cells, with their high energy demand, oxidative burden, and dependence on proteostasis, suggests a corresponding susceptibility, as illustrated by the spontaneous pancreatitis of LAMP-2-deficient mice (Mareninova et al., 2015). For this section, a key contribution of genetically engineered mouse models may lie in reframing cell death in pancreatitis, from a set of independent pathways into an interconnected, switchable network whose configuration determines outcome. The principal experimental models underlying this synthesis, organized by execution pathway and with the corresponding induction protocol, are summarized in **Table 4**.

**Table 4:** Summary of key genetic mouse models investigating regulated cell death in pancreatitis.

RCD pathway	Genetic model/modification	Pancreatitis induction	Ref.
Apoptosis/ Necroptosis	Ripk3−/−; necrostatin-1	Cerulein + TLCS	(Louhimo et al., 2016)
	Ripk3−/− (no protection)	Cerulein	(Newton et al., 2016)
	Ripk3−/− or Mlkl−/− (aggravated)	Cerulein	(Boonchan et al., 2021)
	Ripk3−/−; Atg7 cKO background	Cerulein	(Zhou et al., 2017)
	Saa3−/−	Cerulein	(Yang et al., 2021)
	RIP1 inhibition vs Ripk3 deletion	Cerulein	(Linkermann et al., 2012)
	Venetoclax (pharmacological)	Cerulein; CP	(Litewka et al., 2025)
Pyroptosis	Nlrp3−/−, Asc−/−, Casp1−/−; Tlr9/P2x7−/−	Cerulein; biliary	(Hoque et al., 2011)
	Acinar Nlrp3/Gsdmd cKO; myeloid Gsdmd cKO	Cerulein + bile acid	(Gao et al., 2021)
	Acinar Nlrp3/Gsdmd cKO (HDL)	Cerulein	(Lu et al., 2023)
	Cathepsin B inhibition (pharmacological)	Cerulein	(Wang et al., 2021)
	Pink1−/− or Park2−/−; MCC950	Cerulein; L-arginine	(Zhang et al., 2021)
	PERK knockdown (rat; lentiviral)	Cerulein	(Yan et al., 2023)
	Nlrp3−/−	Duct ligation + cerulein	(Sendler et al., 2020)
	Disulfiram (pharmacological)	SAP	(Zhao et al., 2025)
Ferroptosis	Slc7a11−/−; xCT silencing	Cerulein	(Pan et al., 2023)
	Pancreas-specific Gpx4 KO	Cerulein; L-arginine; ethanol	(Liu et al., 2022)
	Pancreas-specific Arntl (BMAL1) cKO	L-arginine	(Liu et al., 2020)
PANoptosis	Zbp1 knockdown (siRNA)	Cerulein	(Li et al., 2025)
	Eriodictyol (pharmacological)	Cerulein	(Tian et al., 2025)
	Costunolide (pharmacological; endothelial)	SAP	(Ge et al., 2025)
	Qingyi decoction (rat; endothelial)	SAP	(Liu et al., 2026)
	Nano-polyphenol (pharmacological; macrophage)	Cerulein + LPS	(Wu et al., 2026)

## 4 Limitations and future directions

Synthesizing evidence largely from genetically engineered mouse models, this review presents a network view of pancreatitis in which two layers are coupled: a homeostatic network in the acinar cell, comprising digestive-enzyme safety, protein quality control, organelle and autophagy-lysosomal homeostasis, calcium and mitochondrial energy, and NF-κB inflammatory signaling, whose mutually constraining modules determine whether the cell crosses the threshold from health into the injured state; and a downstream execution network in which apoptosis, pyroptosis, necroptosis, and ferroptosis behave not as independent switches but as interconnected, partly interchangeable routes that share molecular nodes such as caspase-8, RIPK3, and GPX4, such that the mode by which a cell dies, and hence disease severity, is shaped in large part upstream by its organellar, metabolic, and energetic state. On this view, severity reflects not any single death pathway but a network configuration rooted in the upstream homeostatic state; the same view helps render the heterogeneity of human pancreatitis intelligible, as diverse genetic and environmental factors enter the same network at different points, and it frames the threshold-dependent, self-perpetuating tendency of injury to spread from a focal event toward the whole gland and the systemic compartment.

Human clinical-genetic data provide independent support for this multigenic, network-based interpretation. In a whole-exome sequencing study of an Indian cohort with chronic or recurrent acute pancreatitis, Prasad et al. reported that every patient carried at least one variant in a pancreatitis-risk gene and that the majority harboured a second variant in an additional risk gene, and they mapped these variants onto shared pathogenic pathways rather than onto a single linear cascade (Prasad et al., 2024). Their two-hit hypothesis, in which the co-occurrence of variants in proteins expressed across both the acinar and ductal compartments is critical for disease, parallels the network view advanced here, whereby diverse genetic lesions enter a common homeostatic network at different points and act in combination to drive the cell beyond its injury threshold. The convergence of an independent human dataset on shared molecular nodes and multigenic causation, derived from patients rather than from mouse models, reinforces the interpretation of pancreatitis as a convergent network failure and identifies these shared nodes as rational, genotype-independent targets for therapeutic intervention.

As a synthetic framework drawn primarily from the evidence of genetically engineered mouse models, the account advanced here carries several boundary conditions that should be made explicit. A first concerns the species difference: mice differ from humans in pancreatic anatomy, genetic constitution, immune system, and disease course, and conclusions drawn from mouse models need not extrapolate fully to human disease. Relatedly, the network framework of this review is to a large extent integrative and explanatory rather than quantitatively validated; the couplings among modules, whether the homeostatic network indeed possesses an additive and modular structure, and the specific links between genotype and homeostatic step and between execution configuration and severity, all currently lack rigorous testing. The model systems carry their own limits: many studies employ single-gene knockouts and a single stress such as cerulein or taurocholate, which isolates variables but does not reproduce the multifactorial, multi-hit, chronic reality of human disease, and the acute-to-chronic transition has not been adequately captured in existing models. The available evidence also derives largely from bulk, tissue-level measurements that can neither distinguish whether the several modes of death occur within the same cell or in different cell subpopulations, nor track switching among death modes in real time; the roles in pancreatitis of PANoptosis, and of recently described modes such as cuproptosis and disulfidptosis, remain for the most part undefined.

These limitations outline several directions for future research. Part of this work bears on testing the framework itself. The heterogeneity of death modes within the acinar population awaits resolution at single-cell and spatial scales: single-cell and spatial transcriptomics can label, cell by cell, the execution program each has engaged, and thereby determine whether marker co-occurrence reflects multi-modal death within one cell or subpopulations proceeding separately toward different termini; reading out the prior metabolic and organellar state in parallel would further test whether the choice of death mode depends, as the framework supposes, on the cell’s pre-existing position in state space. Spatial information is also suited to testing whether dying cells and their newly activated neighbors form discernible gradients or advancing fronts within the tissue, the spatial signature that intercellular DAMP-mediated propagation would predict. Switching among modes, by contrast, calls for temporal resolution: genetically encoded reporters of caspase activity, MLKL oligomerization, lipid peroxidation, and plasma-membrane rupture, combined with intravital imaging of the pancreas or live imaging of organoids, can track within a single cell whether it shifts from one mode of execution to another, helping to test the inference of flux redirection and to clarify whether the choice is committed early or remains plastic. The supposition of an additive, relatively independent modular structure depends on compound perturbation: multiplexed CRISPR editing can alter several loci from different modules within one animal, and comparing the resulting phenotype with the sum of the single perturbations would indicate whether the modules add independently or instead interact synergistically or in a buffering, non-additive manner, turning a qualitative network claim into a measurable coupling structure.

Other directions seek to extend the framework toward human disease and to probe its boundaries. The species difference may be narrowed through humanized models: introducing human pancreatitis loci together with their native regulatory regions into the mouse, by BAC transgenesis or knock-in, would allow the effects of human variants in *PRSS1*, *SPINK1*, *CPA1*, and *CFTR* to be examined against a near-native genomic background, testing whether the proposed genotype-to-homeostatic-step links hold for human alleles; adding humanized immune components would show whether processes peculiar to human disease can be reproduced. Complementary organoid and mouse systems would combine the strengths of each: acinar organoids allow fine-grained mechanistic dissection and rapid genetic manipulation under controlled conditions, while the mouse supplies the integrated immune, vascular, and systemic context together with intercellular propagation; beginning from patient-derived organoids, this complementarity could extend to testing individual modular vulnerability and the propensity toward particular modes of death, offering one route by which the framework might interface with the clinic. The framework further implies that any mode of execution available to the acinar cell may become a terminus in state space, and the acinar profile of high biosynthetic load, high oxidative burden, and particular metabolic dependencies makes it worth asking whether such cells reach the thresholds of metabolically gated modes such as cuproptosis and disulfidptosis; genetic tools directed at the relevant metabolic nodes, such as *FDX1* in cuproptosis, could ascertain their presence and contribution, and so probe whether the execution network extends beyond the modes cataloged to date. Pursued together, these directions trace how a largely explanatory framework can be examined by more quantitative and more disease-proximal study, and revised where the evidence so requires.
